# Empowering Regional Conservation: Genetic Diversity Assessments as a Tool for Eelgrass Management

**DOI:** 10.1111/mec.17656

**Published:** 2025-01-12

**Authors:** Ellika Faust, Kristie Rigby, Anders Olsson, Beatrice Alenius, Per‐Olav Moksnes, Marlene Jahnke

**Affiliations:** ^1^ Department of Fish Ecology and Evolution Swiss Federal Institute of Aquatic Science and Technology (Eawag) Kastanienbaum Switzerland; ^2^ Department of Marine Sciences – Tjärnö University of Gothenburg Stromstad Sweden; ^3^ Department of Marine Sciences University of Gothenburg Gothenburg Sweden; ^4^ County Administrative Board of Västra Götaland Gothenburg Sweden

**Keywords:** conservation genetics, essential biodiversity variables, EU Nature Restoration Law, Kunming‐Montreal Global Biodiversity Framework, population genomics, seagrass

## Abstract

To halt the loss of biodiversity, collaboration among scientists, managers and decision‐makers is vital. Although biodiversity loss is a global problem, management actions influencing diversity are often on a local to regional scale. Our study is an example of a regional conservation genomic assessment developed in collaboration between scientists and managers. We used 2bRAD sequencing to assess 18 eelgrass ( *Zostera marina* ) meadows in northwestern Sweden, an area that has experienced large losses of eelgrass since the 1980s. Genetic diversity was comparable to other assessed meadows in the Atlantic, but an order of magnitude lower than eelgrass in the Pacific. All but one meadow showed high rates of sexual reproduction. Almost all meadows were divergent but grouped into five genetic clusters. Four of the clusters correspond to geographic regions that can be used to define management units. Meadows in areas with a high decline in eelgrass between the 1980s and 2020s are more inbred than meadows in areas with an increase in eelgrass. Overall, our results indicate that striving to protect a number of large eelgrass meadows within each genetic cluster is important for maintaining the genetic diversity and connectivity of eelgrass in northwestern Sweden and is likely beneficial for the wider ecosystem. We estimate current indicators and essential biodiversity variables and discuss their challenges in marine facultative clonal species. We showcase how regional‐scale conservation genomic assessments can serve as a foundation for protection and restoration of a priority species.

## 1 Introduction

In response to the ongoing biodiversity loss, the Kunming‐Montreal Global Biodiversity Framework (GBF) was adopted in 2022, highlighting the urgent need to enhance international cooperation and concerted action to preserve and protect ecosystems (Stephens 2023; CBD 2022a). This includes the global target to protect at least 30% of coastal and marine areas by 2030 and safeguarding species adaptive potential, by maintaining and restoring genetic diversity within and between populations (CBD 2022a). In 2024 the EU Nature Restoration Law was adopted by the European Parliament and the Council of the European Union, which aims to restore at least 20% of sea areas by 2030, and all ecosystems in need of restoration by 2050 (European Parliament 2024). The Nature Restoration Law also requires the development of national restoration and monitoring plans (European Parliament 2024), which are tightly linked to the goals of the GBF (O'Brien et al. 2024). In the GBF, one headline indicator has been agreed on for genetic diversity, stating that effective population sizes (Ne) should be maintained or restored to above 500 (CBD 2022b). This is based on the 50/500 rule of minimum viable populations, which recommends Ne > 50 to minimise short‐term inbreeding and Ne > 500 to allow for populations to adapt in the future (Franklin 1980; Jamieson and Allendorf 2012). Furthermore, Ne can be approximated based on the census population size of mature individuals (Nc). The Ne/Nc ratio varies between species, depending on their reproductive strategies and success (Frankham 1995; Waples 2002). When the Ne/Nc ratio is not known, a ratio of 0.1 is often considered a rule of thumb (Hoban et al. 2021), which makes it an attractive indicator even if genetic data is not available. In addition to the headline indicator of Ne above 500, a complementary indicator was also adopted, ‘the proportion of populations maintained within species’ (CBD 2022b). This indicator is estimated as the number of populations that currently exist, divided by the number of populations that originally existed, and is a measure of whether genetic diversity among populations is maintained (Hoban et al. 2024).

Genetic diversity (see Table 1 for terminology) is in general an essential component for biodiversity as it allows species to adapt to changing environmental conditions and enhances the resilience of species to diseases, and other threats (Booy et al. 2000). It ensures that populations have the necessary variation to adapt, ultimately supporting ecosystem stability and functioning (Hughes et al. 2008). International conventions (CBD 2022a) increasingly recognise the value of genetic diversity and population structure assessments in relation to conserving biodiversity. The Group on Earth Observations Biodiversity Observation Network (GEO BON) has developed Essential Biodiversity Variables (EBVs) to help evaluate how aspects of biodiversity vary over time and space. One of the six EBVs are the ‘genetic composition EBVs’, which offer practical means to monitor spatial and temporal variability in intraspecific genetic composition, genetic health and viability (Hoban et al. 2022). The four genetic EBVs include: (i) genetic diversity, the richness and evenness off diversity among individuals, (ii) genetic differentiation, the number of populations and their degree of divergence, (iii) effective population size, the number of individuals in an idealised population that will exhibit the same amount of genetic diversity loss as the population under consideration, and (iv) inbreeding, the mating between related individuals (Hoban et al. 2022). The genetic EBVs are also being explored and applied in a genetic monitoring program of temporal trends of genetic diversity by the Swedish Agency for Marine and Water Management (SwAM) (Johannesson and Laikre 2020; Kurland et al. 2024; Ries et al. 2023; Saha et al. 2024), but have rarely been applied to species in the marine environment where a high proportion of species have ‘unusual’ life‐histories.

**TABLE 1:** Terminology commonly used in conservation genetics/genomics.

Term	Explanation
Genetic differentiation	The evolutionary change/difference in allele frequencies among populations
Genetic connectivity	Transfer of genetic material (via floating shoots or seeds) among populations
Genetic diversity	The amount of genetic diversity within and among population(s). Sometimes referred to as intraspecific diversity
Heterozygosity	Fraction of heterozygotes in a population, directly estimated from individual genotypes
Locus	The position on a chromosome of a gene or marker. Here defined as a SNP
Allele	Alternative form of a gene or a marker
SNP	Single nucleotide polymorphism is a single nucleotide site in a DNA sequence that is polymorphic and has two alleles. SNPs can be used as a marker to assess genetic diversity
Polymorphic sites (Pol)	The number of sites in a population where some variability is observed, that is, more than one allele is present
Private alleles (Pa)	An allele that is present in only one of many populations sampled
Genotypic diversity	Sometimes also referred to as clonal diversity in clonal plants; it stands for the number of different genotypes within populations, that is, is the opposite of clonality. Higher genotypic diversity is seen as something to strive for
Genotypic richness (R)	Similar to genotypic diversity but corrected for sample size
Clone	Genetically—nearly—identical groups of organisms originating from the same individual through asexual reproduction
Multilocus genotype (MLG)	An organism's genetic composition (set of alleles) across multiple loci
Multilocus lineage (MLL)	Multilocus lineages are a proxy for clonal lineages which rely on the computation of genetic distances between pairs of MLGs to distinguish organisms belonging to the same clonal lineage from organisms of different lineages. MLLs allow the identification of clonal lineages despite missing data, genotyping errors or mutations occurring in any one clone
Inbreeding	The mating of individuals that are closely related through common ancestry
Runs of homozygosity (ROH)	Inbreeding generates homozygote segments along the genome called runs of homozygosity (RoH). The length of RoH is directly related to the number of generations to a common ancestor and these RoH can be used to estimate an organism inbreeding, known as FRoH
Effective population size (Ne)	Effective population size (Ne) is an evolutionary parameter introduced by Sewall Wright (Wright 1931), described as the size of an ideal population (constant size, random mating and non‐overlapping generations) that would experience the same amount of genetic drift as the observed population
Restoration	The process of assisting the recovery of a degraded, damaged, or destroyed habitat or ecosystem

Seagrasses are marine flowering plants which occur in both temperate and tropical seas and play important roles in coastal areas for ecosystem functioning and carbon sequestration (Short et al. 2007). With seagrass being able to process carbon 30 times faster than rainforests, they play a crucial part in the global ecosystem (Serrano et al. 2011). Eelgrass ( *Zostera marina* ) is the most common seagrass species in the Northern hemisphere (Short et al. 2007; Boström et al. 2014) and can reproduce clonally via its rhizomes, and sexually using negatively buoyant seeds attached to floating reproductive leaves. The reproductive leaves can disperse for tens to hundreds of kilometres and may then release seeds which in turn sink to the seafloor (Källström et al. 2008; Jahnke et al. 2018). Eelgrass is a foundation species for shallow water ecosystems (Duffy et al. 2022) with a number of critical ecological functions, including enhancing the recruitment of fish and crustaceans (Bertelli and Unsworth 2014), water quality (Reusch et al. 2021) and the sequestration of carbon and nitrogen (Röhr et al. 2018). Furthermore, eelgrass is most likely the best‐studied species of any plant or animal for which evidence of the large impact of genetic diversity on ecosystem functioning and associated species exists. Many previous studies on eelgrass have shown that high genotypic diversity (i.e., the number of genetically different clones in a given sample) is associated with increased resistance (Hughes and Stachowicz 2004), resilience (Ehlers, Worm, and Reusch 2008; Reusch et al. 2005) and productivity (Hughes et al. 2008; Gross et al. 2024).

Similar to other seagrass species eelgrass has seen a decline along its entire distribution, mainly driven by anthropogenic impact and climate change (Waycott et al. 2009; Boström et al. 2014). Along the Swedish northwest coast approximately half of eelgrass has vanished since the 1980s (Baden et al. 2003; Moksnes and Bergström in press), with up to 90% decline in some regions (Moksnes et al. 2018). The loss of eelgrass also results in the loss of several important ecosystem services (Nordlund et al. 2024). For example, the loss of 10 km^2^ of eelgrass along the Swedish north west coast was estimated to have resulted in a release of 60,000 tons of carbon and 6600 tons of nitrogen stored in the sediment, resulting in a total cost to society of 141 million US$ (Moksnes et al. 2021).

Previous studies combining conservation genetics with biophysical modelling have identified valuable and vulnerable eelgrass meadows along the Swedish west coast on both large and small scales within fjords (Jahnke et al. 2018, 2020). These and other larger‐scale studies suggested that genetic diversity along the Swedish west coast is comparable to meadows assessed in the Northeast Atlantic (Jahnke et al. 2018; Ries et al. 2023; Duffy et al. 2022; Yu et al. 2023). The probability of dispersal of reproductive shoots with ocean currents is also relatively high (Jahnke et al. 2018), and biophysical dispersal models suggest that floating shoots can disperse up to 100–200 km (Jahnke et al. 2018). However, the probability of dispersal is reduced in some areas featuring high rates of decline (Jahnke et al. 2020) and high rates of clonality (Ries et al. 2023). These results highlight the importance of protecting existing meadows and give hope for local recovery, if decline can be halted (Jahnke et al. 2020). From these initial assessments, the Swedish Agency for Marine and Water Management has initiated temporal genetic monitoring of eelgrass across the entire distribution in Sweden (Ries et al. 2023). This genetic monitoring with a high value for detecting and halting the loss of biodiversity (Ries et al. 2023; Kurland et al. 2024; Saha et al. 2024) and required under the CBD's global biodiversity framework (GBF) makes Sweden one of the pioneers in temporal genetic monitoring in Europe (Pearman et al. 2024). Eelgrass is also considered in several EU directives and conventions focusing on the marine environment and biological diversity (EU 2008).

In Sweden, the responsibility for selecting protected areas, as well as managing nature reserves and supervising activities impacting the environment, is carried out by the different county administrative boards (CABs) or less commonly the municipalities (Holmgren, Sandström, and Zachrisson 2017). Regional and national managers consider eelgrass a priority species when establishing protected areas under national and international environmental goals. Since the formation of marine protected areas (MPA) is costly and time‐consuming, it is important that the resources are deployed where they do the most good. For managers, the genetic mapping of eelgrass is therefore a very important piece of the puzzle to be able to evaluate the connectivity between existing MPAs and plan the location of future MPAs to strengthen the network. Therefore, even one‐time (rather than temporal), regional‐scale genetic assessments are particularly applicable. When research and management practices are combined, assessments of genetic diversity and connectivity can immediately be incorporated into the challenging task of prioritising meadows that are either especially vulnerable or valuable for the entire metapopulation (Riginos and Beger 2022; Jahnke and Jonsson 2022).

Thus, there is a need for improved knowledge of eelgrass meadows on a regional level for the CABs of Sweden to be able to make relevant management decisions. Consequently, this study was commissioned by the CAB of Västra Götaland, with funding from the Swedish Agency for Marine and Water Management. Using 2bRAD sequencing and well‐established sampling methods we investigated the four genetic EBVs: (i) genetic diversity, (ii) genetic differentiation, (iii) effective population size and (iv) inbreeding from 18 eelgrass meadows in the region. As a facultative clonal species was assessed, we also discuss what implications clonality may have on the genetic EBVs and their usefulness as indicators for marine species that switch between reproductive modes. We also assessed clonal diversity as a fifth potential variable. The presented information currently directly aids management decisions on a weekly basis, such as selecting protected areas, granting exemptions and sourcing donor material for restoration in the region.

## 2 Material and Methods

### 2.1 Sampling

**FIGURE 1:**
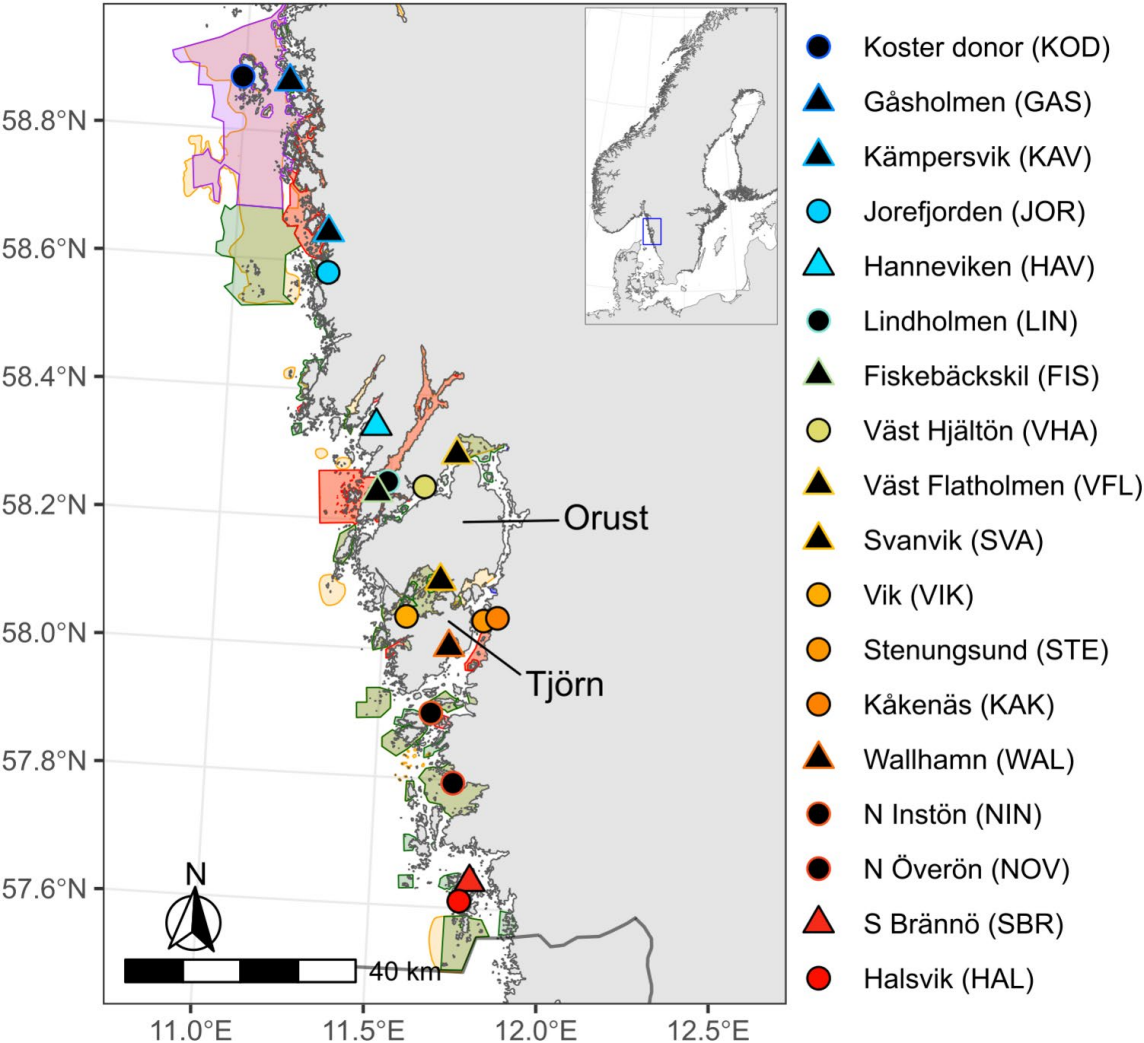
Map of protected areas and the 18 assessed eelgrass meadows along Västra Götaland in Sweden. Meadows are shown as points where colours go from blue in the north to red in the south. Reference meadows are shown as circles and impacted meadows as triangles. Small meadows (< 20 ha) have a black fill and coloured lines and large meadows have colour fill and black edges. Protected areas displayed include Natura 2000 areas (orange), nature reserves (green), national parks (purple), biotope protected areas (blue) and nature conservation areas (red). Note that different types of protection can and do overlap.

We used a sampling design—most notably with an approximate distance of 1.5 m among sampled shoots—which has been used in many global studies (Yu et al. 2023; Duffy et al. 2022) and is identical to the one used in previous assessments in Sweden (Jahnke et al. 2018, 2020; Ries et al. 2023). At each of the 18 assessed eelgrass meadows, 20 shoots were collected (Figure 1, Table 2, Table S1). We designed the sampling to have geographically close paired bays, where one meadow was ‘impacted’ based on the degree of physical disturbance from recreational boating activities and constructions (e.g., docks, piers, dredging channels etc.), and a nearby ‘reference’ meadow. Meadows were classified as impacted if ≥ 30% of the shoreline or bottom area in the bay showed signs of physical disturbance and classified as a reference area if < 5% showed signs of physical disturbance. Reference meadows were not as likely to be exposed to physical impact from boating but could still be exposed to other stressors. One temporal assessment was also included by re‐sampling the meadow Stenungsund (STE) which was collected in 2020, at a distance of 500 m at Kåkenäs (KAK) in 2022. Meadows were classified as ‘small’ when < 20 ha, and ‘large’ when > 20 ha.

**TABLE 2:** Information on the 18 assessed eelgrass meadows, see Figure 1 for a map of the different meadows.

Waterbody	Meadow	Code	Size ha	Impact	nInd	nMLL	Cluster
N Bohusläns skärgårds kust	Koster donor	KOD	7.5	Reference	17	8	C1
N yttre Tjärnöarkipelago	Gåsholmen	GAS	NA	Impacted	20	11	C3
Fjällbacka inre skärgård	Kämpersvik	KAV	2.7	Impacted	20	13	C1
Fjällbacka inre skärgård	Jorefjorden	JOR	169.4	Reference	19	16	C1
Brofjorden	Hanneviken	HAV	34.2	Impacted	19	2	
Getevikssund	Lindholmen	LIN	10.2	Reference	20	12	C1
Gullmarn centralbassäng	Fiskebäckskil	FIS	1.9	Impacted	20	18	C2
Koljö fjord	Väst Hjältön	VHA	31.6	Reference	20	17	C2
Kalvöfjord	Väst Flatholmen	VFL	0.4	Impacted	20	13	C3
Stigfjorden	Svanvik	SVA	1.9	Impacted	20	12	C4
Stigfjorden	Vik	VIK	51	Reference	20	14	C4
Hake fjord	Stenungsund	STE	47.1	Reference	20	16	C5
Hake fjord	Kåkenäs	KAK	47.1	Reference	20	16	C5
Hake fjord	Wallhamn	WAL	6.7	Impacted	19	13	C5
Älgöfjorden	N Instön	NIN	6.4	Reference	15	13	C5
Nordre Älvs fjord	N Överön	NOV	4.2	Reference	13	9	C5
Brännö‐ Styrsöområdet	S Brännö	SBR	55.2	Impacted	19	19	C5
Halsviken	Halsvik	HAL	34.5	Reference	20	11	C5

*Note:* We assessed both reference and impacted meadows in this assessment—particularly with the aim to detect future changes in genetic diversity. Meadows categorised as ‘reference’ have no larger ‘physical impact’ (e.g., docks and marinas). Cluster indicates to which genetic cluster the meadows are assigned to (see Figure 2).

Abbreviations: nInd, number of individuals; nMLL, number of unique multilocus lineages.

#### 2.1.1 DNA Extraction and Library Preparation

Directly after sampling and cleaning, eelgrass leaves were transferred into a portable freezer and kept at –20°C. Eelgrass was then freeze‐dried and genomic DNA was extracted from ca. 20 mg of freeze‐dried sample using the Macherey‐Nagel NucleoSpin plant II kit following the manufacturer's instructions. DNA extractions were standardised to 50 ng per reaction for the digestion step in the 2bRAD library protocol (https://github.com/z0on/2bRAD_denovo). This was followed by ligation and amplification following the original protocol. Overall, DNA extractions and library preparations were prepared for 419 samples (including replicates) from 18 meadows and sent for sequencing at SciLifeLab in Sweden on an Illumina NovaSeq 6000 platform. The same method was also used in genetic monitoring by SwAM (Ries et al. 2023), and the two datasets were analysed in conjunction following the established pipeline. This strengthened the analysis by adding a larger number of samples and more technical replicates.

### 2.2 Bioinformatic Processing

Raw reads sequenced on different lanes were concatenated and quality checked with fastqc and were subsequently analysed in conjunction with raw reads from Ries et al. (2023) to allow for better comparison between the data. Reads were then processed following the 2bRAD GATK pipeline (https://github.com/z0on/2bRAD_denovo). First a filter was applied to only keep reads with a match to the restriction site, removing pcr‐duplicates. Poor quality ends (Q < 15) were trimmed using cutadapt ‘4.4’ (Martin 2011). Trimmed reads with a minimum length of 25 bp were then mapped with the local option in bowtie2 ‘2.5.1’ (Langmead and Salzberg 2012) to the *Z. marina* genome v.3.1 (Ma et al. 2021). Once mapped, read groups were added and files were converted, sorted and indexed using samtools ‘1.17’ (Danecek et al. 2021). Bam files were validated with Picard ‘2.23.4’ ValidateSamFile. Variants were called with the Genome Analysis Toolkit (GATK 3.8; McKenna et al. 2010) UnifiedGenotyper and subsequently filtered for a minimum depth of 3. Technical replicates were used to produce training data of true variants for variant quality score recalibration using a custom Perl script with a minimum of one heterozygous pair. Some technical replicates were excluded from the training data set due to high missingness and poor heterozygous match (≤ 0.9), leaving a total of 60 technical replicates for recalibration. The recalibrated data set was filtered to only keep bi‐allelic sites with a maximum missingness of 10%, maximum heterozygote fraction of 0.5 and individuals with a missingness below 40%. SNPs occurring on the same RAD‐tag were thinned, leaving only the SNP with the highest minor allele frequency. Finally, individuals with a missingness above 25% were removed.

### 2.3 Clonality

Assessing clonality of genomic data is not straightforward, as both sequencing errors and somatic mutations will cause two clones to have slight differences in their sequences. To overcome this problem, we included 2–4 duplicate individuals per meadow (technical replicates) for which library preparation and sequencing were carried out twice independently. Clones were then grouped based on genetic distance where each group can be considered a unique multilocus lineage (MLL; Ries et al. 2023). The threshold of genetic distance to consider two genotypes unique was evaluated using the R package poppr ‘2.9.3’ (Kamvar, Tabima, and Grünwald 2014). To ensure the robustness of MLL assignment, distances were calculated using both bitwise and Euclidean distance, and thresholds were estimated using three clustering algorithms based on maximum distance, minimum distance and the average distance (UPGMA) between pairs. This was then compared to the maximum distance between the technical replicates, ensuring all technical replicate pairs fell within the same MLL. After this, technical replicates were removed and the data was clone‐corrected, keeping one unique genotype from the different MLLs in each meadow.

### 2.4 Population Structure and Divergence

Population assignment was done using three different individual clustering approaches on the clone‐corrected data. Individual ancestry coefficients were estimated using a sparse non‐negative matrix factorisation algorithm (sNMF) in the R Package LEA ‘3.6.0’ (Frichot and François 2015). The ancestry coefficients represent the proportions of each individual's genome that originated from a specified number of ancestral populations K, allowing for estimates of mixture between populations. Individual ancestry was estimated for K 1–20 in 20 separate runs and the best K was evaluated both visually and with the cross‐entropy criterion. Individual assignment was also estimated using discriminant analysis of principal components, DAPC (Jombart, Devillard, and Balloux 2010) in adegenet ‘2.1.10’, with grouping based on the best K from LEA. Finally, individuals were clustered using a principal component analysis (PCA) with the R package ade4 ‘1.7.22’ (Chessel, Dufour, and Thioulouse 2004; Dray, Dufour, and Chessel 2007), which makes no prior assumptions about how many populations exist or boundaries between them. Allele frequencies were centred but not scaled and missing data were replaced by mean allele frequencies with the function scaleGen in adegenet (Jombart 2008; Jombart and Ahmed 2011). When comparing genetic diversity values among genetic clusters, individuals from the same meadow were all assigned to the cluster with the highest overall assignment. We estimated genetic divergence between meadows with pairwise F_ST_, and significance using dartR ‘2.7.2’ (Gruber et al. 2018; Weir and Cockerham 1984). *p*‐values were corrected for multiple testing using the false discovery rate correction (Benjamini and Hochberg 1995). Divergence and distribution of variation were assessed with an analysis of molecular variance (AMOVA) among the five clusters, among meadows and within meadows using the R package poppr (Kamvar, Tabima, and Grünwald 2014). We tested for isolation‐by‐distance by analysing the relationship between pairwise genetic distance, F_ST_/(1 − F_ST_), and geographical distance among the different meadows. Geographical distance was estimated as shortest waterway distance with no depth restriction, with the package marmap ‘1.0.9’ (Pante and Simon‐Bouhet 2013). If meadows were erroneously considered to be on land due to grid size, the coordinates were slightly adjusted to be in the water. The relationships between pairwise individual genetic distances and in‐water geographic distances were tested with a Mantel test and visualised with dartR.

### 2.5 Genetic Diversity

Genetic diversity was estimated on the clone‐corrected data for each meadow. Genotypic richness was calculated with the following formula (MLL − 1)/(nInd − 1). Private alleles were calculated with the package poppr (Kamvar, Tabima, and Grünwald 2014). Heterozygosity, F_IS_ and number of polymorphic sites were calculated with the package dartR (Gruber et al. 2018). Nucleotide diversity was estimated with vcftools ‘0.1.16’ (Danecek et al. 2011). The correction factor (2 × nMLL)/(2 × nMLL − 1) was applied to compensate for the underestimation of genetic diversity estimates due to sample size, similarly as done by dartR for unbiased heterozygosity (Gruber et al. 2018).

Contemporary estimates of effective population size (Ne) were estimated for each meadow with the newly developed program currentNe v1.0 (Santiago et al. 2024). CurrentNe uses linkage disequilibrium to estimate Ne and confidence intervals. The software also corrects for non‐independence among SNPs using chromosome number and for complex mating systems by assessing and including full siblings in the data. Ne estimates were then used to calculate the Ne/Nc ratio for each meadow. In a recent survey of eelgrass in the region, the average shoot density was estimated as ca 550 shoots per m2 (Moksnes and Bergström In press). However, there can be many shoots on one plant. Assuming 10 shoots per plant, we multiplied the meadow size by the average number of plants (55) to calculate Nc, which was then used to calculate the Ne/Nc ratios. We also produced a second estimate to somewhat correct for the level of clonality at a meadow by multiplying Nc with genotypic richness and then calculating the ratio as Ne/Nc_MLL_.

Individual runs of homozygosity (ROH; continuous stretches of DNA where the genetic material is identical on both chromosomes) were calculated using all SNPs before removing monomorphic sites with the sliding window approach in PLINK 1.9 (Purcell et al. 2007) and the –homozyg function. Parameters were set to a minimum ROH size of 300 kb, a maximum gap of 400 and a minimum density of 7 SNPs per window and 10 SNPs per ROH. To account for genotyping errors 1 heterozygous site and 2 missing calls were allowed per window, and a maximum of 1 heterozygous call per ROH. Genome coverage, proportion of the genome that ROH can cover given the SNP density (Meyermans et al. 2020), was estimated by converting all SNPs to homozygous and dividing the total length of all ROH by the genome size (240.2 Mb), excluding scaffolds smaller than 300 kb. Individual inbreeding coefficients (FRoH) were estimated for ROH > 1 Mb and ROH > 5 Mb by dividing the total length of ROHgenome size (FRoH_1Mb_: 229.6 Mb, FRoH5_Mb_: 228.4 Mb).

To investigate the genetic consequences of eelgrass decline, we correlated genetic diversity estimates, inbreeding and effective population size with changes in meadow size and eelgrass areal distribution in coastal water bodies (the management unit used in the European Water Framework Directive and generally at the scale of a fjord or similar) since the 1980s (Moksnes and Bergström in press). Correlations were done in R with the function ggscatter from the ggpubr ‘0.6.0’ package (Kassambara 2023). We did this analysis separately for the data set presented here, as well as including additional 24 meadows previously assessed with microsatellites in Västra Götaland (Jahnke et al. 2018, 2020). To be able to compare different assessments with microsatellites and SNPs, genetic diversity was classified as low, medium and high based on either the percentage of polymorphic alleles (SNPs) or allelic richness (microsatellites) for the combined analyses. Genotypic richness estimated from microsatellites and SNPs was directly correlated without transformation. For those meadows in our assessment for which information on meadow size existed (12 meadows) we also correlated the change in meadow size with genetic diversity at the meadow.

Genetic diversity estimates were summarised by taking the average for reference and impacted meadows, for small and large meadows and for each genetic cluster identified with the above population assignment approaches. Effective population size was also estimated for each cluster separately. Pairwise *t*‐tests were done to estimate if there was a significant difference between impacted and reference meadows as well as between small and large meadows. Unless otherwise stated, data manipulation and visualisation of results were done using R v4.1.2 (R Core Team 2021) and Rstudio ‘2024.4.1.748’ (Posit team 2024) with the Tidyverse ‘2.0.0’ package (Wickham et al. 2019).

## 3 Results

DNA extraction, library preparations and sequencing were successful for 341 of 360 plants, with a minimum of 13 plants from each meadow (Table S1). A total of 25,761 SNPs were called, and 12,444 were bi‐allelic where 8900 had a missingness below 10%. Of these, 8792 were polymorphic and had a heterozygosity which did not exceed 0.5. After thinning to one SNP per RAD‐tag, 5849 SNPs remained, of which 4864 were polymorphic among the 404 samples which passed filtering (including 63 technical replicates). Among the 341 samples, there were a total of 232 MLLs (Figure S1A–C, Table S3). The number of MLLs stayed the same irrespective of distance estimates or algorithm used and all technical replicates clustered within the same MLL. One MLL contained individuals from two geographically distant meadows Gåsholmen (GAS) and Väst Flatholmen (VFL), five and nine shoots, respectively. No other clones were shared among any of the other meadows. The clone corrected data set thus consisted of 233 individuals and 232 MLLs, where one MLL was present in two different meadows (Table S3).

### 3.1 Genetic Differentiation

Pairwise F_ST_ estimates were < 0.11 (Table S4), but significant among all sites except VFL and GAS, which were not significantly divergent and also shared an MLL. Genetic divergence between meadows did show a significant correlation with geographic distance (*R* ^2^ = 0.136, *p* = 0.004), suggesting that isolation‐by‐distance plays a significant role in genetic divergence between the meadows (Figure S2).

**FIGURE 2:**
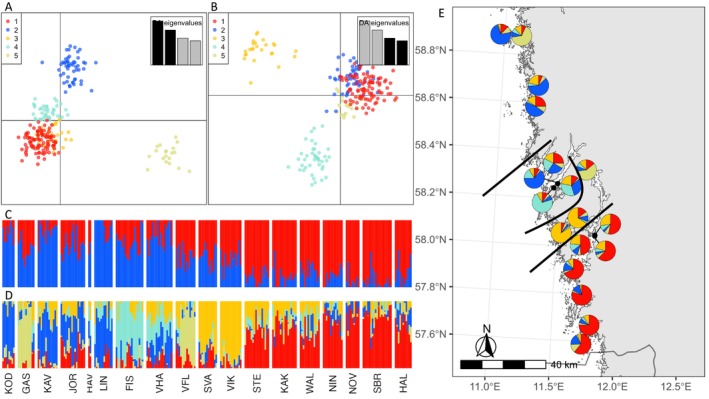
Genetic clustering analysis displaying principal components of DAPC for axis 1–2 (A) and axis 3–4 (B). The analysis of genetic ancestry with LEA showing patterns for 2 (C) and 5 clusters (D and E). Meadows are ordered from North to South. Every vertical line represents one individual and the colour shows the proportion of each individual assigned to each of the genetic clusters. (E) The genetic clustering analysis summarised as pie charts and projected onto a geographic map, showing patterns for K = 5 clusters, as well as the approximate locations of barriers to gene flow between the four geographically distinct clusters (black lines).

To identify the most likely number of K clusters, we used LEA, DAPC and visual inspection. All clustering methods display a similar pattern of divergence. In the PCA, the first axis mainly separates southern and northern individuals (Figure S3). The second axis displays additional variation within these two groups, with Lindholmen (LIN), Fiskebäckskil (FIS) and Koster (KOD) being the most differentiated. On the third and fourth axis Gåsholmen (GAS) and Väst Flatholmen (VFL) cluster together and separate from the rest (Figure S3). The lowest cross‐entropy estimated by LEA, and thus the best model fit, was K = 13, but also K = 5 and K = 10 displayed a clear dip in cross‐entropy criterion, indicative of a good model fit (Figure S4). In contrast, the lowest BIC value used by DAPC to find the optimal number of clusters was K = 1, thus not supporting any additional clusters (Figure S5). Based on these estimates and visual inspection, the K = 5 cluster was selected as the optimal solution, displaying clear clusters, with a reduced risk of over‐splitting. Both DAPC and LEA were used to assign meadows to the different clusters (K = 5), and to the northern or the southern clusters (K = 2). The two assignment methods were in agreement for all but two meadows (Figure 2; Table S5). Hanneviken (HAV), where only two individuals remained after clone‐correction, was assigned to different clusters for both K = 2 and K = 5 and was thus excluded. Väst Flatholmen (VFL) was assigned to the Southern cluster by LEA and to the Northern by DAPC and was thus excluded at K = 2 assignment (Table S5). The AMOVA also showed significantly less variation within meadows and more variation between meadows and the five clusters compared to what would be expected under the null hypothesis (Table S6).

The analysis of genetic ancestry with LEA displayed clear patterns of differentiation and many hierarchical levels of K (Figure 2; Figure S6). At K = 2 there was a clear difference between northern and southern meadows, where most individuals in the northern parts assign predominantly to the blue cluster and individuals in the south to the red cluster, while those in between assign equally to both (Figure 2C). The separation between the North and South meadows was equally distinct on the first discriminant axis of DAPC (Figure S7). At K = 5 admixture shows clear genetic clustering into four geographic areas from North to South as well as a fifth cluster consisting of Gåsholmen (GAS) and Väst Flatholmen (VFL; Figure 2). These latter two meadows also share an MLL. These same clusters can be observed in the four discriminant axes retained in the DAPC (Figure 2A,B). Higher K values displayed additional substructure within the five clusters, most often clustering adjacent meadows together (Figure S6).

### 3.2 Genetic Diversity

**FIGURE 3:**
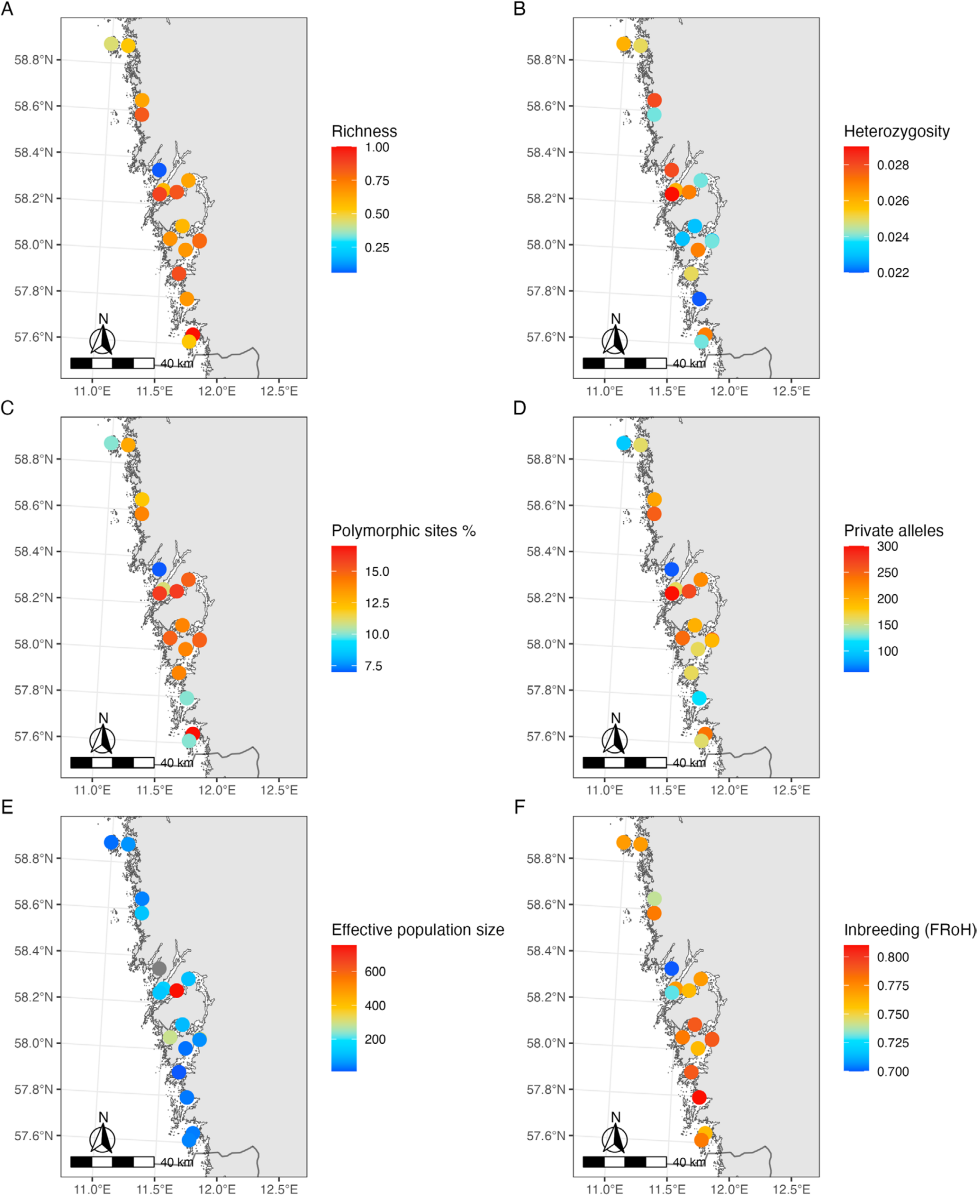
Mapping of genotypic and genetic diversity of 18 eelgrass meadows in Västra Götaland. (A) Genotypic richness (the reverse of clonality). 1 indicates that each sampled individual had a unique genotype. 0 indicates that all 20 sampled shoots belong to the same clone; (B) observed heterozygosity; (C) percentage of polymorphic SNPs per meadow and (D) number of private alleles, that is, genetic variants that were exclusively found at one meadow; (E) effective population size Ne; (F) inbreeding calculated from runs of homozygosity. Grey points are NA where diversity has not been assessed due to too small sample size after clone correction.

On the regional level, genotypic richness was high, particularly in comparison to eelgrass meadows in the Swedish Baltic Sea (Ries et al. 2023). However, the meadow at Hanneviken (HAV) had low genotypic richness with only two unique MLLs (Table S1; Figure 3A). Koster (KOD) and Halsvik (HAL) also had high levels of clonality. The only meadow sampled which did not have any clones was S Brännö (SBR). Mean genotypic richness varied among the genetic clusters from 0.58 to 0.87 (Table S1, Figure S8). Small and large meadows were comparable (Table S1, Figure S9A), but genotypic richness tended to be lower at impacted vs. reference meadows (impacted: 0.62, reference 0.70, Table S1, Figure S9B).

Observed heterozygosity and nucleotide diversity were overall low across all meadows (Ho = 0.022–0.029, *π* = 0.022–0.027), and different groups (Table S1; Figure 3B). Almost all meadows also displayed a negative F_IS_, indicating heterozygosity excess (Table S1). The number of polymorphic sites was relatively few in all meadows (uPol = 313–835), with the highest being S Brännö (SBR) and the lowest Hanneviken (HAV; Figure 3C). Small meadows had on average less (ca. 7% less) polymorphic sites than large meadows but with high variability among meadows (Table S1). When comparing clusters, the Northernmost cluster 1 had the lowest number of polymorphic loci, and cluster 2 the highest (Table S1). Levels of private alleles in contrast were generally high (Table S1; Figure 3D). Small meadows had on average fewer private alleles than large meadows (Figure S9). The meadow sampled at the temporal scale of two years (STE and KAK) had very similar genotypic and genetic diversity estimates at both occasions (Table S1).

The largest effective population (Ne) size, and the only above 500, was found in Väst Hjältön (VHA; Ne = 754; Table S1; Figure 3E). Only two other meadows, Stenungsund (STE) and Vik (VIK), had Ne confidence intervals which overlapped 500. Effective population size varied widely within the five genetic clusters and was on average more than 60% smaller in impacted vs. reference and small vs. large meadows (Table S1, Figure S9B). Ne estimates for clusters remained small, and none had confidence intervals which overlapped 500. Calculations of Ne/Nc gave overall low ratio estimates and ranged between 0.000001 and 0.000918. Ratios remained low when correcting for genotypic richness and ranged between 0.000001 and 0.00145 for Ne/Nc_MLL_.

Estimates of runs of homozygosity (ROH) suggested good genome coverage (average 81%) and most ROH were in the range of 1–5 MB, but several runs were above 10 MB (Figure S10). Individual inbreeding coefficients (iFRoH_1Mb_) were generally even and ranged from 0.59 in FIS to 0.87 in NOV (Figure S11). On average per meadow, Hanneviken (HAV) had the lowest inbreeding (0.70) and Svanvik (VIK) and N Överön (NOV) had the highest (0.81; Figure 3F). FRoH_5Mb_ estimates based only on large ROH (> 5mb) showed similar even patterns with values running from 0.19 to 0.34, with also the highest values in NOV and the lowest in FIS (Figure S11).

**FIGURE 4:**
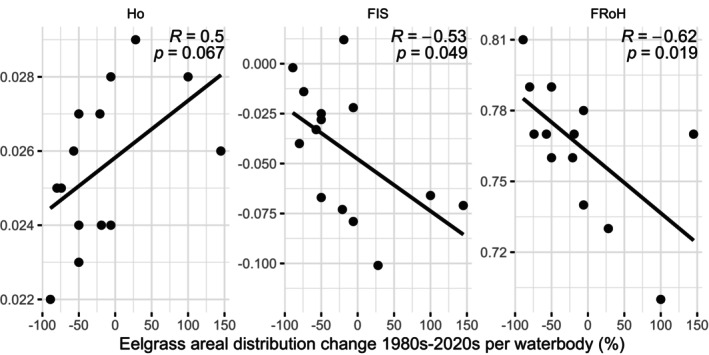
Correlation between three genetic diversity estimates (F_IS_, FRoH and Ho) and overall change in eelgrass areal distribution between 1980s and 2020s as percentage per waterbody.

The analysis of the overall change in eelgrass areal distribution within water bodies showed a significant negative correlation with inbreeding (both FRoH_1Mb_ and F_IS_; Figure 4; Figure S12). Similarly, observed heterozygosity and effective population size showed a positive correlation, yet not significant using the Pearson correlation coefficient. However, both heterozygosity and effective population size were significant when testing with the Spearman correlation coefficient, suggesting a positive, but non‐linear correlation (Figure S12B). The estimated change in meadow size did not show any significant correlations with any genetic diversity estimates (Figure S13). Genetic diversity estimates were also not significantly different between small and large meadows (Table S1). Between impacted vs. reference meadows genetic diversity (Ho and Pi) was higher and inbreeding (FRoH_1Mb_) was lower in impacted meadows. Although we did not see any significant correlations when combining the data with previous microsatellite analyses (Jahnke et al. 2018, 2020), meadows with low diversity indicated a negative correlation between genotypic richness and change in waterbody eelgrass areal distribution (Figure S14), which suggests that meadows with low genetic diversity may rely more on clonal growth during meadow expansion.

## 4 Discussion

Biodiversity loss is one of the largest catastrophes of our time, and within species diversity is increasingly being recognised for its importance for species survival. Although biodiversity loss is a global problem, regional knowledge is needed for effective conservation actions. Protected areas and other conservation actions are often managed by counties, municipalities or other regional or local offices, but conservation genomic assessments are at best scarce at this regional level, particularly in the marine environment. Here we assessed the genetic composition of eelgrass in the county of Västra Götaland in northwestern Sweden with four genetic Essential Biodiversity Variables: (i) genetic diversity, (ii) genetic divergence, (iii) effective population size and (iv) inbreeding. We also added a fifth diversity measure, which is important for facultatively sexual species: genotypic richness, which is the inverse of clonality. We also discuss the challenges of working with a facultative clonal species when interpreting the genetic EBVs as well as the CBD indicators for genetic diversity (the proportion of populations within species with an effective population size > 500 and proportion of populations maintained within species; CBD 2022b). We summarise the main recommendations based on our results in Box 1 and discuss, from a scientist and a manager perspective, how these results can be and are used for protection and restoration of eelgrass.

BOX 1 **Concrete recommendations for the management of eelgrass in the region of Västra Götaland (VG region) based on this genetic assessment.**

**Table:** 

Locality	Finding	Recommendation/conclusion/outcome
VG Region	Sexual reproduction is frequent	Clonality is not generally a concern for protection and restoration in Västra Götaland
VG Region	Genetic diversity is low, but comparable to the low diversity in entire Atlantic	Maintenance of genetic diversity is especially important
VG Region	Isolation by distance (IBD)	In most cases, geographically close meadows are also genetically similar and can thus provide good donor material for restoration. It is important to maintain or re‐establish meadows at a distance that allows dispersal and stepping‐stone connectivity over many generations
VG Region	Almost all meadows are genetically different, but overall they can be grouped into 4 clear genetic clusters	Use the genetic clusters as management units and ensure that 30% is protected within each genetic cluster. Avoid using donor material for restoration from a different genetic cluster. Ideally, make a genetic assessment of potential donor meadows for any restoration action
VG Region	Estimates of effective population size (Ne) are generally very small and inbreeding overall high	Maintenance of genetic diversity and connectivity is especially important for this species, for example, protection, restoration, facilitating gene flow, and genetic monitoring
Impacted vs. Reference	Physically impacted and reference meadows have similar levels of genetic diversity and inbreeding. Ne is lower at impacted meadows	Physical disturbance appears to have had little impact on genetic diversity. Meadows impacted by physical disturbance can therefore be used as donor material for restoration. Ne may be an early indicator of impact/decline in eelgrass due to physical disturbance
Small vs. Large	Small meadows have lower genetic diversity, are more inbred and have a lower Ne. Declining meadows have higher levels of inbreeding	It is important to preserve the areal distribution of eelgrass and especially large meadows, and aim to increase the area of smaller meadows, for example, through restoration. It is better to use donor material from large meadows for restoration, as they are more likely to harbour eelgrass with higher genetic diversity
S Brännö	Has high genotypic richness and a high percentage of polymorphic alleles	Particularly valuable due to its high genetic diversity. May represent good donor material for restoration efforts in the southern cluster
Väst Hjältön, Stenungsund, Vik and Svanvik	Are stepping stones between the Northern and Southern clusters	Particularly important to protect eelgrass in these areas around Orust to ensure connectivity. All four assessed meadows are already under area‐based protection
N Överön	Fragmented meadow that is the only remaining eelgrass in the Ryskärsfjord area after > 95% loss. Has low diversity and shows signs of inbreeding	Very vulnerable. The meadow is in a protected area but may need additional actions, including restoration and assisted gene flow
Hanneviken	Consists of only two genotypes	Do not use as donor material in restoration because of high clonality
Gåsholmen and Väst Flatholmen	The same clone was detected at these two meadows more than 100 km apart	Do not use either meadow as donor material, as it seems likely that eelgrass has been transferred artificially

### 4.1 Essential Biodiversity Variables and Protection Measures

For managers, the most important information from this study is that eelgrass meadows in the region are genetically divergent, but can be grouped into distinct genetic clusters (Box 1). The four main clusters are larger than the defined waterbodies, the unit used in ecological status assessment for the Water Framework Directive and MPA design. Meadows sampled in the same waterbody always assigned to the same genetic cluster. Waterbody may therefore be a good unit for eelgrass management (Jahnke et al. 2020). When aiming for connected networks of MPAs (Figure 1), it may be beneficial to rely on the larger genetic clusters, as well as allowing for dispersal within and among clusters (Box 1). An estimated 38% of all meadows in the region are currently in protected areas (Figure 1; Länsstyrelsen Västra Götaland 2020), and the next step would now be to ensure that > 30% of meadows in each genetic cluster are protected.

While 100 s–1000 s meadows remain genetically unassessed in Västra Götaland alone, our analysis also highlights some vulnerable and valuable meadows in the region. One example of an especially valuable meadow is Brännö with particularly high genetic diversity (Box 1). This meadow itself is not currently under protection, but other eelgrass meadows close by (1–10 km) are in protected areas. In the last decades, consideration of eelgrass has been high when exploitation cases such as recreational docks have been handled by authorities, both inside and outside MPAs, and has shifted even further towards considering eelgrass conservation and genetic diversity. Nevertheless, docks, marinas and other infrastructure with older permits must be allowed to function, including maintenance of dredged channels.

High clonality is considered to render a population more vulnerable to environmental change (Procaccini, Dattolo, and Ruocco 2023; Ries et al. 2023; Johannesson 2024). Most meadows had a heterozygosity excess, indicative of clonality (Reichel et al. 2016; Arnaud‐Haond et al. 2020). The only meadow with really high clonality was Hanneviken. While the shoots at Hanneviken and the meadow itself looked very healthy and flowering was observed during sampling, this meadow is right next to Sweden's largest oil refinery. Nevertheless, eelgrass in the waterbody Brofjorden has increased by 100% and Hanneviken itself has increased by 42% in size since the 1980s (Moksnes and Bergström in press). When combining our data with previous microsatellite data, we observed a pattern that expanding meadows with low diversity are more clonal than declining meadows. We suggest that meadows with low diversity may thus rely to a larger extent on clonal reproduction when expanding compared to more diverse meadows where no such difference could be observed (Figure S14). Moreover, change in eelgrass areal distribution per waterbody was significantly correlated with inbreeding in the SNP data set (Figure 4). Without pinpointing causation this indicates that eelgrass decline may be associated with inbreeding depression.

The only meadows where Ne estimates, or their confidence intervals, exceeded the viable population threshold of 500 were three large reference meadows. This validates the current strategy of the CAB to protect large eelgrass meadows. Environmental impact can generate demographic changes, which with time can lead to a decline. However, it can take several generations before a decline in genetic diversity can be observed, known as time lag or extinction debt (Gargiulo, Budde, and Heuertz 2024). While physical impact is certainly a threat to eelgrass (Eriander et al. 2017), the relationship between impact and genetic diversity in facultatively clonal seagrasses is further complicated, as disturbance may increase the rate of sexual reproduction (Alexandre, Santos, and Serrão 2005; Jahnke, Olsen, and Procaccini 2015). This could also explain why impacted meadows had slightly, but significantly, higher genetic diversity (Ho and Pi) and lower inbreeding (FRoH_1Mb_). Overall, it is clear that striving for protection of a number of large eelgrass meadows within each genetic cluster is important for the goal of maintaining genetic diversity and connectivity of eelgrass in Västra Götaland. In areas where large eelgrass losses have occurred, it may also be appropriate to protect the seabed where eelgrass has already disappeared to ensure that they can recover naturally or be restored. In Sweden, protecting eelgrass started to become important in the mid‐1980s. As more MPAs are being established, population structure identified in genetic studies is considered in management of the MPAs, often on a weekly basis, to ensure future gene flow, genetic diversity and adaptive potential.

### 4.2 Indicator Assessment

Only one meadow had an estimated Ne above 500, which would give an estimate of 0.06 for the Ne > 500 indicator. However, if the genetic clusters identified are used, the Ne > 500 indicator is 0, because none of the clusters has Ne above 500. Effective population size estimates below the threshold of 500 used for viable population size assessments (CBD 2022b), have been observed commonly in plants (Clarke et al. 2024). While partial clonal reproduction should not significantly impact effective population size, unless sexual reproduction is extremely rare, larger and higher frequency clones may contribute more to the next generation, thereby reducing Ne (Balloux, Lehmann, and de Meeûs 2003). Small sample size and admixture can also show downward bias estimates of effective population size (Neel et al. 2013; Ryman, Laikre, and Hössjer 2019; Gargiulo et al. 2023, 2024). However, our estimates of Ne by genetic clusters—with larger sample sizes and less admixture—still show Ne < 500, corroborating the small effective population sizes of eelgrass in the region. Low effective population sizes below 500, or even below 50, may be natural for some species without negative impacts, for instance species with partial clonal reproduction (Hoban et al. 2024; Clarke et al. 2024). For these species, tracking Ne and census population size (Nc) over time may provide more insight into population changes than relying on a single point estimate. Hence, the applicability of the 50/500 rule and the 500 Ne cut‐off for eelgrass and other partially clonal species may need further research, and more emphasis should be on temporal monitoring of changes in Ne and other genetic diversity estimates.

A recent assessment of historic losses of eelgrass found that over 1000 ha of eelgrass have been lost since the 1980s, equivalent to 45% of the eelgrass in the region (Moksnes and Bergström in press). Despite the large decline, complete loss of all eelgrass in a bay is rare. If we define loss as a reduction of > 99%, only 18 bays out of the 164 bays assessed would be considered lost. This would give an estimate of 0.89 proportion of populations maintained. Thus, we propose, also to report the percentage of areal distribution maintained in sessile species such as eelgrass.

In recent years there has been a lot of development in mapping eelgrass using aerial drones, which allows for temporal monitoring of eelgrass beds relatively inexpensively (Huber et al. 2022; Berglund et al. 2023). In theory these estimates of meadow size could then be used to estimate and track changes in Ne. However, the rule of thumb of 0.1, often considered a conservative estimate for many species (Hoban et al. 2021; Clarke et al. 2024), is far from suitable for estimating Ne in eelgrass. Our results suggest that census size of eelgrass far exceeds the effective population size (Ne/Nc ~0.00007 and Ne/Nc_MLL_ ~0.0001) and that Ne/Nc ratio is in need of being further evaluated, both in this and other facultative clonal species. Many of the genetic diversity indicators are strongly built on population genetic theory of Hardy Weinberg equilibrium, recombination, or rely on considerable levels of diversity, and our findings highlight the need for further research into the applicability of genetic indicators for facultative sexual organisms.

### 4.3 Restoration Measures

In the past two years, major changes in marine environmental policy have been made (Sutherland et al. 2023). The CBD agreement is to protect 30% of marine areas by 2030 (Target 3 GBF) and to maintain and restore genetic diversity of native wild and domesticated species (Goal A and Target 4 GBF). The EU Nature Restoration Law mandates the restoration of 30% of degraded marine habitats by 2030, 60% by 2040 and 90% by 2050. Importantly, seagrass is mentioned specifically as a habitat type in Annex II of the EU Nature Restoration Law (European Parliament 2024). There is clear evidence that genetic diversity plays a crucial role in the success of restoration efforts. Conservation genomic approaches can be used to contribute to restoration by developing concrete advice aimed at restoring biodiversity and population viability (van der Valk and Dalèn 2024), but also as a tool to assess the effectiveness of restoration efforts (O'Brien et al. 2024).

Previous studies have shown that genotypic diversity correlates with successful seagrass restoration (Reynolds et al. 2012; Evans, Vergés, and Poore 2017). Moreover, the genetic variability and origin of donor meadows have an important influence on the success of seagrass restoration (Reynolds et al. 2012; Jahnke, Olsen, and Procaccini 2015). This conservation genomic assessment of 18 meadows along a coast of 150 km, and with a median distance of 53 km among assessed meadows, is a sufficient resolution for incorporating genetics in a relevant way in management and protection. However, finer resolution will often be necessary for restoration. Knowledge of genetic diversity influences the prioritisation of restoration efforts and is an important basis for selecting donor meadows and should be included in species and habitat restoration manuals. We suggest choosing donor meadows from the same genetic cluster with high genetic diversity in the local context, to avoid the introduction of less favourable genetic material. The CAB of Västra Götaland is in the process of adding the genetic information from this study to eelgrass maps to aid management, for example, when deciding on environmental permits and licences.

Since the results of the genetic assessment became available in 2023, the CAB of Västra Götaland is taking the genetic clusters into consideration in management decisions on a weekly basis. For instance, genetic differentiation shown in Figure 2 was taken into account when choosing a donor meadow for a pilot restoration planting south of Stenungsund. In this case donor material with local adaptation was desirable, and a donor meadow within the same genetic cluster was chosen. Looking ahead, funding has been granted for the planting of three hectares of eelgrass south of Stenungsund. The restoration site is troubled by high turbidity and resuspension of sediment, causing bad light conditions. To improve light conditions sand capping of at least five hectares of sea bottom will be conducted before eelgrass is planted. An important future direction for seagrass restoration would therefore be to assess potential adaptation to low light conditions within each cluster.

The research on local adaptation and genomic vulnerability (Pazzaglia et al. 2021; van der Valk and Dalèn 2024), is still in the stage of basic rather than applied research. Furthermore, forecasting species' responses to shifting environmental conditions (Seaborn et al. 2021) and predicting individual fitness and maladaptation (Rellstab, Dauphin, and Exposito‐Alonso 2021) in a given environment based on genotype (Lotterhos 2024) will play an important role in restoration efforts in the face of climate change (Wood et al. 2020). Selecting donor material with pre‐adaptive traits to future environmental conditions, known as predictive provenancing (Breed et al. 2019)—instead of aiming to bring back exactly what was lost—may be the only way to ensure long‐term success of restoration efforts. To really take advantage of these recent developments, very fine‐scale genetic assessments around every potential future restoration meadow would be ideal, in order to find the best genetic fit of donor material (considering clonality, genetic differentiation, genetic diversity and adaptive traits) for any given current and future environmental condition.

#### 4.3.1 Science‐Management Collaboration

The Swedish guidelines for eelgrass restoration was published in 2016 (Moksnes et al. 2016). In the last years several large‐scale (> 1000 m^2^) restoration projects have been completed around the Swedish coast and more are planned in the near future (Gagnon et al. 2023, Moksnes et al. in press). The knowledge of genetics could therefore be applied quite early on in the history of eelgrass restoration in Sweden. One of the challenges in restoring habitats and species is securing long‐term funding, something which is hard to come by both for managers and academic researchers. Funding and political priorities change over time which is something managers and scientists alike have to adapt to. The increased area‐based protection and stronger focus on maintaining genetic diversity through the commitments made within the GBF and the new EU Nature Restoration Law may provide some stability in funding and are likely to greatly alleviate direct threats to biodiversity.

For the overall aim of including genetic results in protection and restoration actions, it is important that administrators and case handlers are informed about the results of genetic studies, trained in how they can be used, and ideally involved in the planning. Officials at regional and local level rarely have access to scientific articles and do not have time to keep up to date with the latest research (Sandström et al. 2019). On the other hand, scientists are normally not informed about the needs of the managers, for example, what type of scientific knowledge and data they need for handling local cases or in reporting for different EU legislations and international conventions. Conservation genomics face an additional issue since genetic results cannot currently be reported under any European legislation. While the Marine Strategy Framework Directive had originally the indicator *1.3.2 Population genetic structure* under D1, this has been removed in the first revision after no country reported on this indicator in the first assessment. This lack of a standardised way of reporting prevents the use of genetics by different stakeholders (Leigh et al. 2024). However, there is hope that the monitoring under the new EU Nature Restoration Law may provide an opportunity for standardised reporting of genetic diversity to the EU (O'Brien et al. 2024). If genetic data is made available and case handlers at authorities know which populations are genetically valuable or vulnerable, it can have important consequences for the assessment in small‐scale exploitation cases and permit matters, for example, dredging applications for a marina. This knowledge and competence are also needed when assessing compensation cases where eelgrass is to be moved or restored. When dealing with small‐scale exploitation cases and other permit matters, the cumulative effects of completed and ongoing exploitation in an area should be considered (Eriander et al. 2017), particularly on the level of genetic clusters.

The above‐mentioned limitations appear to pose a serious impediment to the application of genomic methods in marine management, as we are not aware of any similar efforts of using conservation genomics in a science‐management collaboration on a regional scale for eelgrass or marine species in general. In our experience, the key to the successful use of genomic methods in the present study was a trustful and long‐standing collaboration between individual managers and scientists resulting from working together for many years on, for example, national guidelines for eelgrass restoration and management (Moksnes et al. 2016; Moksnes, Larsson, and Tullrot 2017), which was partly promoted by the Swedish Institute for the Marine Environment (SIME), a collaboration between six Swedish universities with the task to facilitate the transfer of knowledge between academia and marine management. However, to succeed in implementing conservation genomics on a larger variety of species and situations, the collaboration between universities and CABs must increase, which remains mainly based on individual relations. Improving the communication falls on both parties and is necessary for marine management to move forward.

## 5 Conclusion

The loss of biodiversity is a global issue, but the type and level of threats vary across areas, and so will the roads to solutions. International agreements, consortia and collaborations are imperative to share knowledge, set goals and to hold ourselves accountable. We must then follow through with decisions and actions on a national and local level. It is crucial to recognise that we will need to work on many scales and use a diverse set of approaches to manage and conserve biodiversity across the globe. To develop effective conservation actions, we need to define what geographic scale makes sense from a biological and environmental perspective, but also from a governance and management perspective. We provide here a detailed example on how conservation genomic assessments on a spatial scale relevant to management are applied in conservation and restoration of a priority species. We show that conservation genomic technology and knowledge are now firmly established and can be directly translated into management actions, and were able to apply all four suggested genetic biodiversity variables to this marine non‐model species. It is both feasible and affordable for governments and local authorities to incorporate genomic tools, indicators and information into wider monitoring, protection and restoration programs.

## Author Contributions

M.J., P.‐O.M., A.O. and B.A. designed the research. E.F. and K.R. performed the research. E.F. and M.J. analysed the data. All authors wrote the paper. Writing was led by E.F. and M.J.

## Conflicts of Interest

The authors declare no conflicts of interest.

## Supporting information


Figure S1.–S14.

Table S1.–S6.

## Data Availability

The vcf file that supports the findings of this study is openly available at https://doi.org/10.5061/dryad.r2280gbnv and the bioinformatic scripts and 2bRAD walkthrough used were taken from https://github.com/z0on/2bRAD_denovo. Additional code is available on github https://github.com/ellikafaust/Zostera‐marina‐vg. All raw sequences are available on the NCBIs Sequence Read Archive (BioProject PRJNA1043091).
