# Supplementary material for: Empowering Regional Conservation: Genetic Diversity Assessments as a Tool for Eelgrass Management
Source: Mol Ecol. 2025 Jan 12;34(23):e17656. doi: 10.1111/mec.17656 (PMC12684364; doi:10.1111/mec.17656)

**Empowering Regional Conservation:
Genetic diversity assessments as a tool for eelgrass management**

Ellika Faust, Kristie Rigby, Anders Olsson, Beatrice Alenius, Per-Olav Moksnes, Marlene Jahnke

**Table of Contents:**

[**Figure S1: Visualisation of multilocus lineages, MLLs.**](#_xrtp61f3nvp2) **2**

[**Figure S2: Isolation-by-distance**](#_mnah24u3qicc) **5**

[**Figure S3: Principal component analysis (PCA)**](#_4cz0d9uk8bde) **6**

[**Figure S4: cross-entropy criterion**](#_nlsvjda8wa6) **7**

[**Figure S5: BIC values**](#_m51z91ttpxw5) **8**

[**Figure S6: Genetic clustering analysis**](#_9l8wmoie4k5j) **9**

[**Figure S7: DAPC density plot.**](#_7ng9tpx6jns0) **10**

[**Fig S8: Cluster genetic diversity estimates**](#_jhfzhsey4am6) **11**

[**Fig S9: Meadow genetic diversity estimates**](#_2dm9aomy4t6l) **12**

[**Fig S10: ROHs**](#_p2607xmt6aqr) **14**

[**Fig S11: FRoH**](#_8bc62rv1izpo) **15**

[**Fig S12: Diversity and distribution correlation**](#_4q11yags6npc) **16**

[**Fig S13: Diversity and size correlation**](#_vxpi0vju2seu) **18**

[**Fig S14: Richness and waterbody change**](#_amaktat6mxaw) **19**

# Figure S1: Visualisation of multilocus lineages, MLLs.

*A) The number of MLLs for different genetic distance cut offs for each of the three algorithm. B) Tree based on euclidian genetic distances with cut-off thresholds for each algorithm visualised (8.4, 8.7 and 8.9) as well as the largest distance between any two technical replicates (7.6). The colour indicates different MLLs, with unique MLLs in grey. C) Visualisation of the number of MLLs per meadow. For instance, at Koster (KOD), we found eight different MLLs. One of these was present 7x (MLG402), three were present two times each, and the remaining four clones were only unique.*

**A)**

*
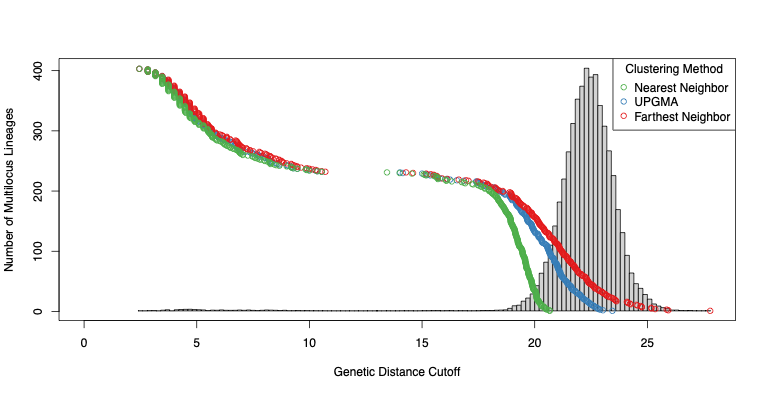
*

**B)**

*
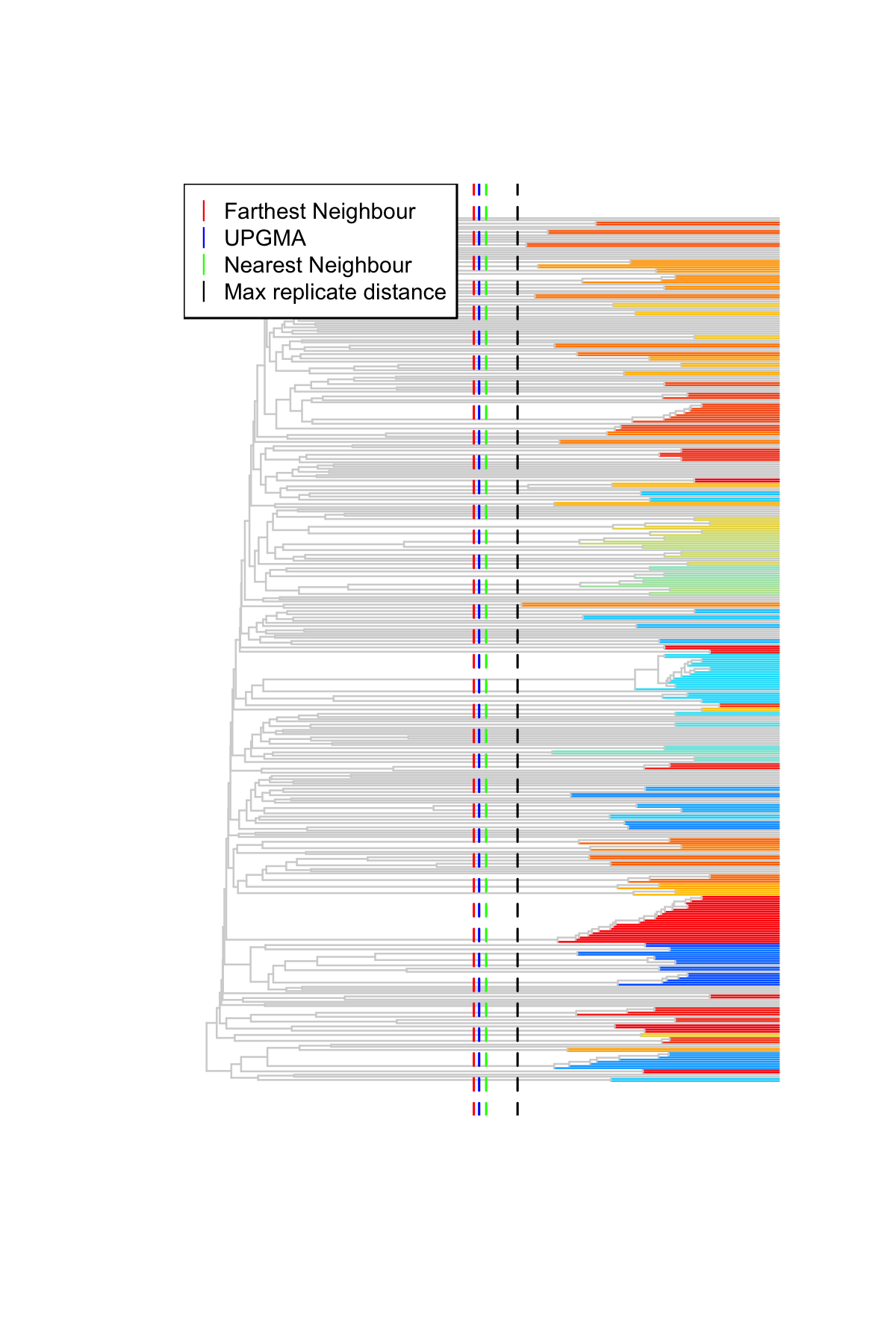
*

**C)**


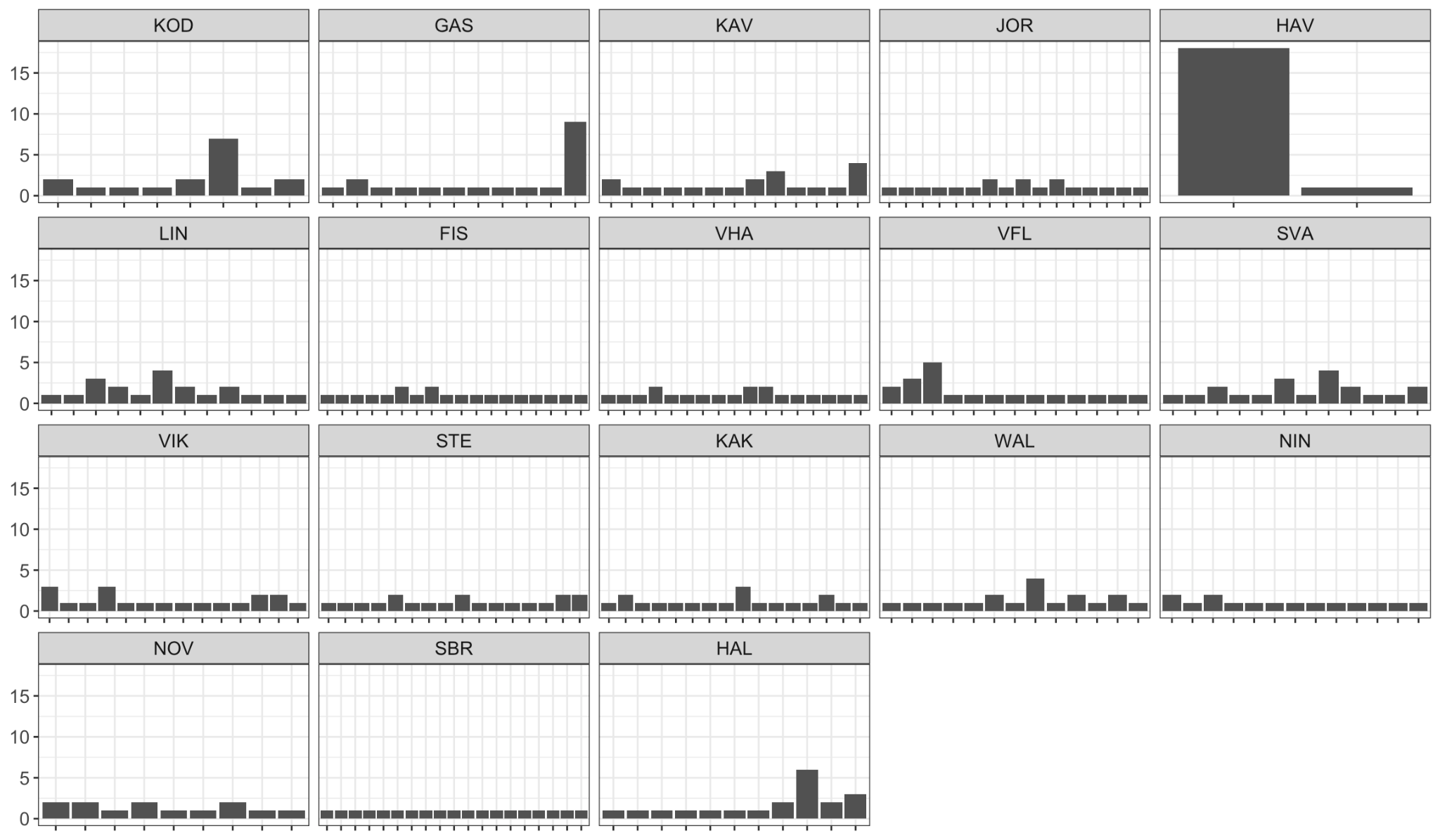


# Figure S2: Isolation-by-distance

*Isolation-by-distance correlation plot of pairwise genetic distance (FST/(1-FST)) and sea distance among the different meadows. Sea distance was estimated as the shortest waterway distance with no depth restriction. If meadows were erroneously considered to be on land due to grid size, the coordinates were slightly adjusted to be in the water. The relationships between pairwise individual genetic distances and sea distances was tested with a Mantel test.*


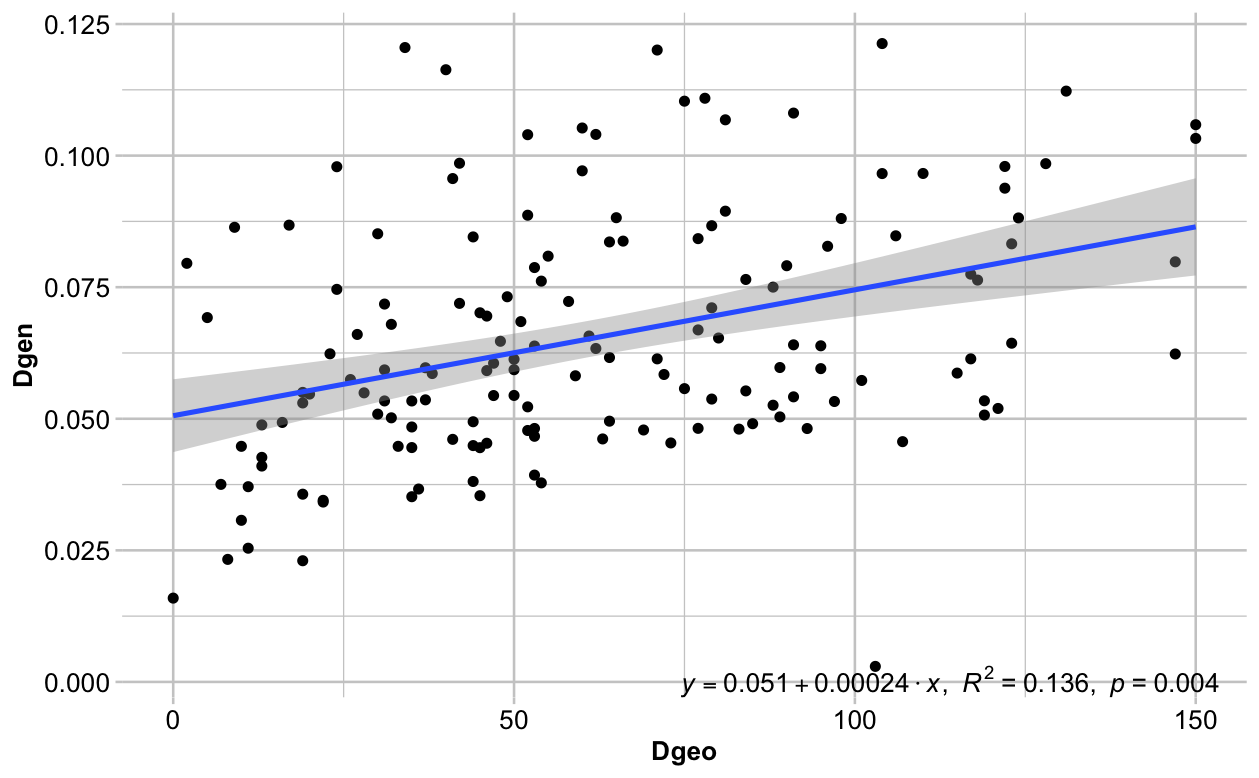


# *Figure S3:* *Principal component analysis (PCA)*

*Principal component analysis (PCA) for additional principal components of the 18 eelgrass meadows. Each meadow has its symbol as explained in the legend. Northern meadows are coloured in shades of blue, and more Southern meadows are in shades of red. The location of each dot represents the genetic makeup of an individual in relation to all assessed individuals. Similar individuals cluster closely together. The eigenvalues of each axis are displayed in the bottom right corner.*

*
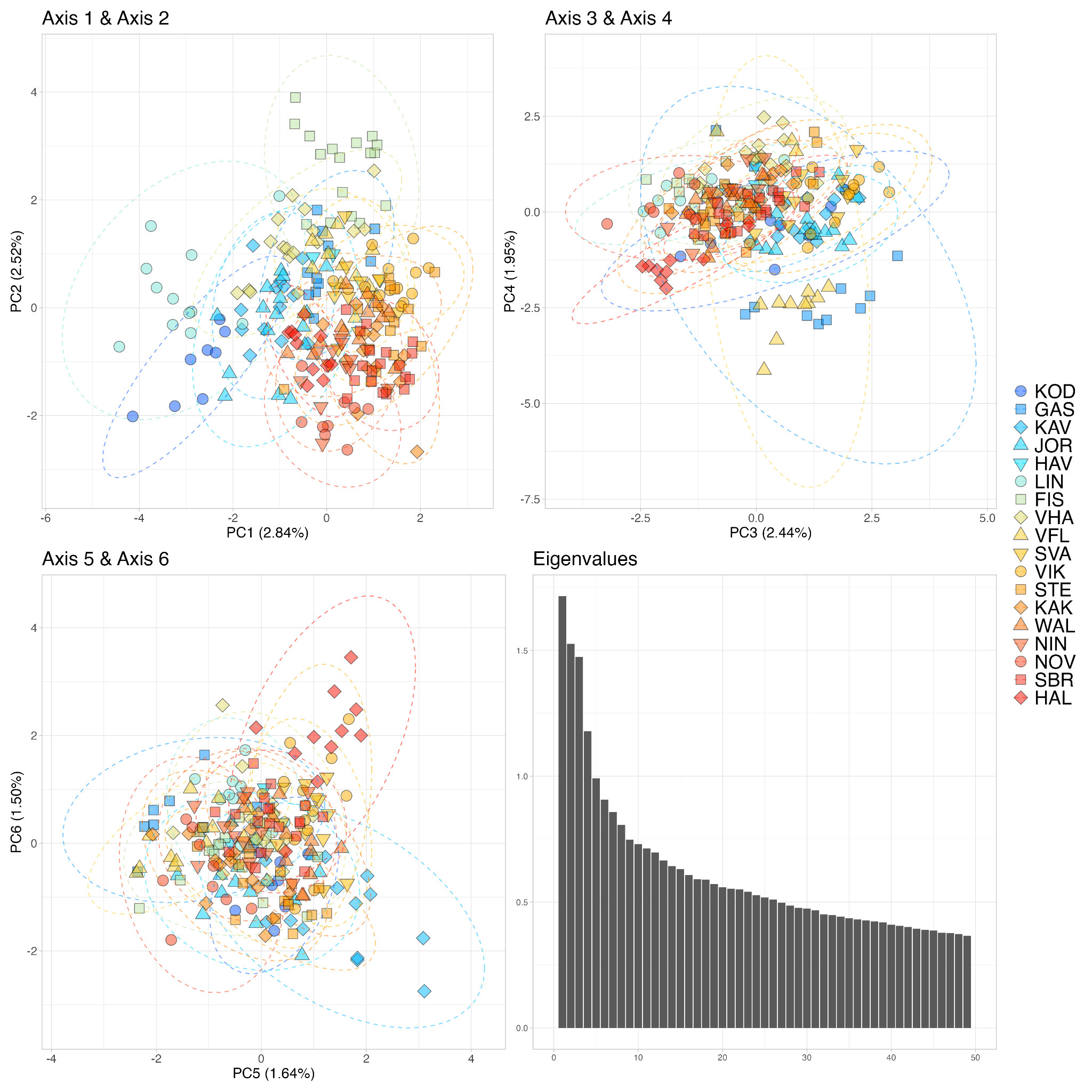
*

# Figure S4: cross-entropy criterion

*Values of the cross-entropy criterion as a function of the number of factors used in snmf() runs from the R package LEA.*


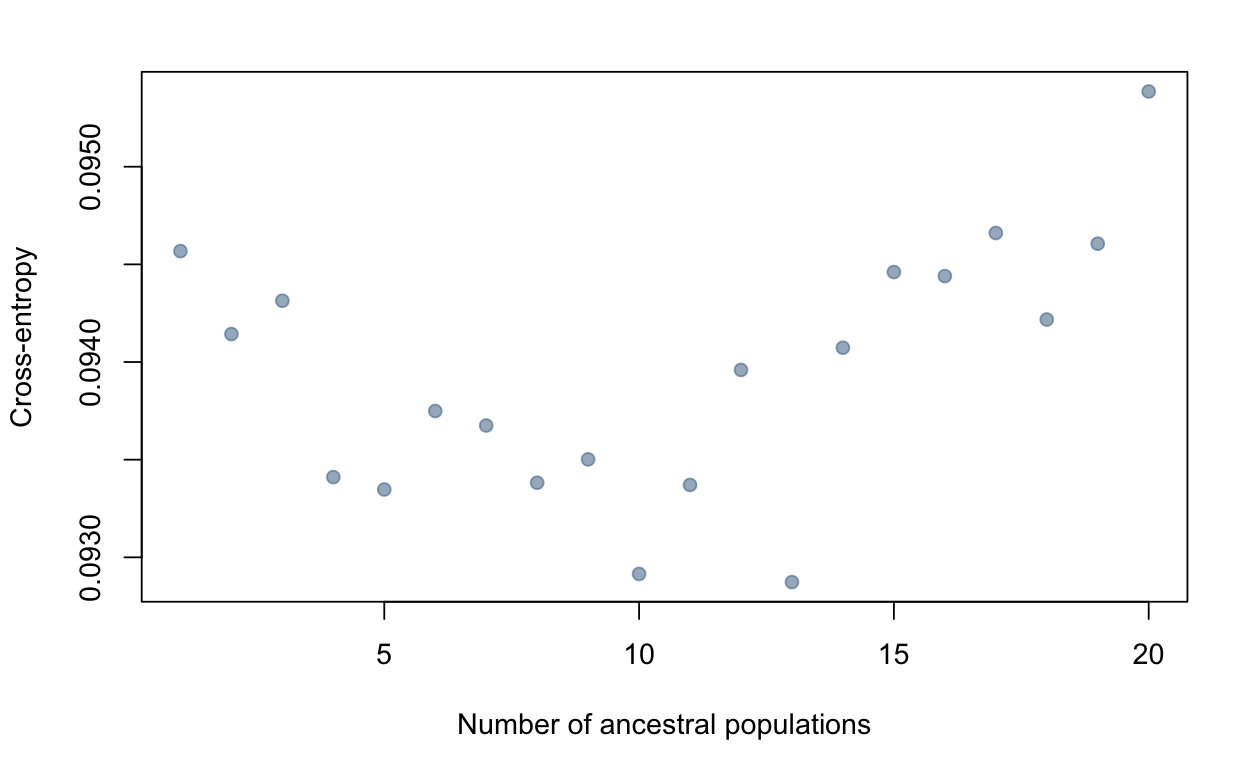


# Figure S5: BIC values

*BIC values versus the number of K clusters evaluated by DAPC.*


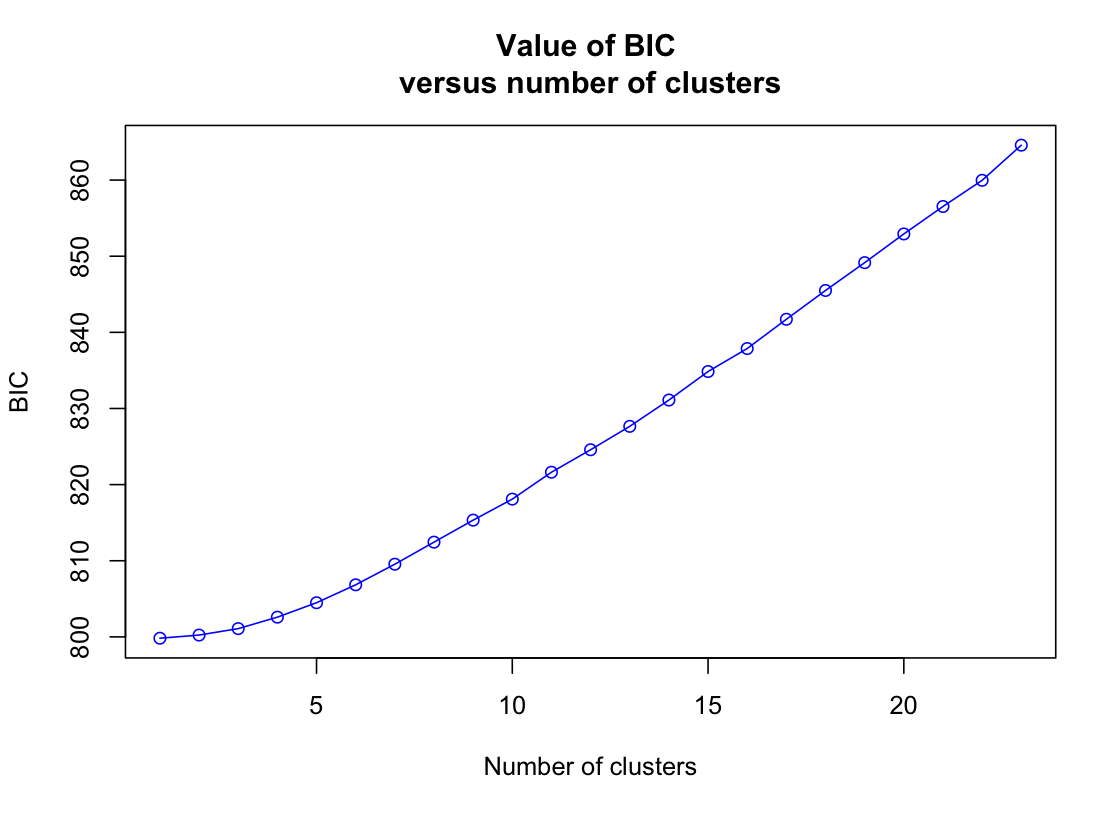


# Figure S6: Genetic clustering analysis

*Genetic clustering analysis, showing patterns for K = 2-10 clusters. Meadows are ordered from North to South. Every vertical line represents one individual and the colour shows the proportion of each individual assigned to each of the genetic clusters.*
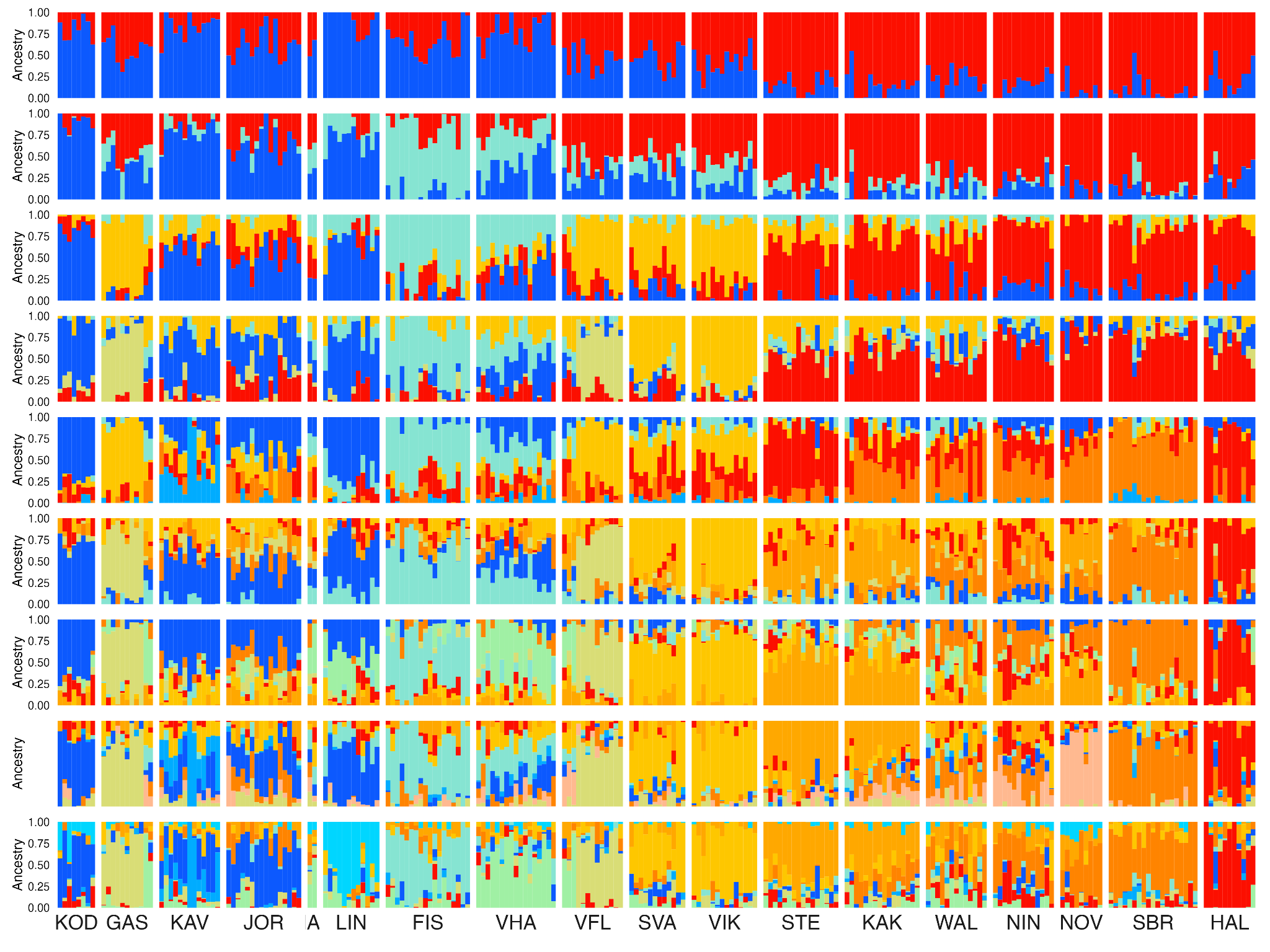


# Figure S7: DAPC density plot

*Density plot of individuals along the first discriminant axis in the DAPC. The top two plots show the individuals coloured by assignment and the bottom shows their meadow origin*

​​
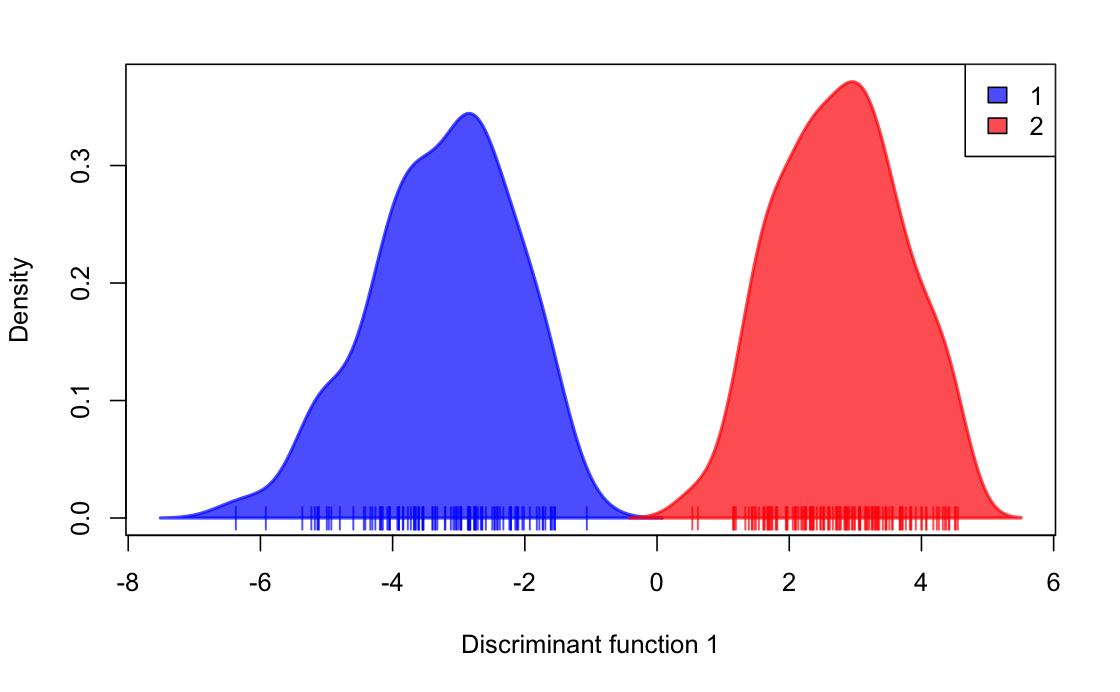

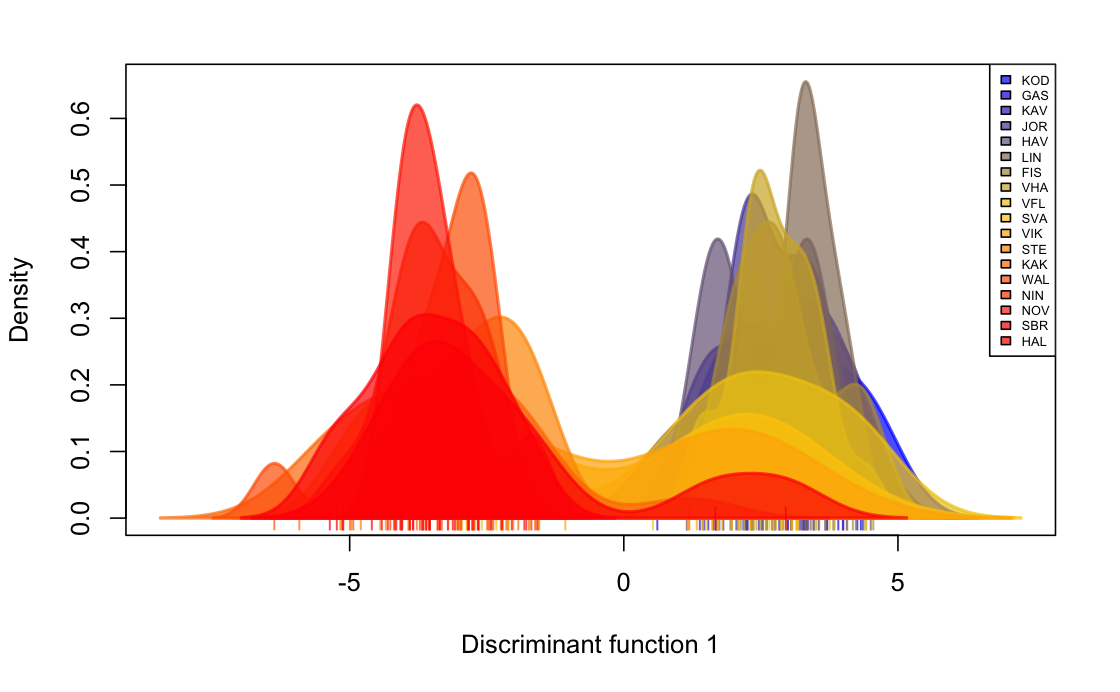


# Fig S8: Cluster genetic diversity estimates

*Mean genetic diversity estimates for the five clusters identified with DAPC and admixture.*


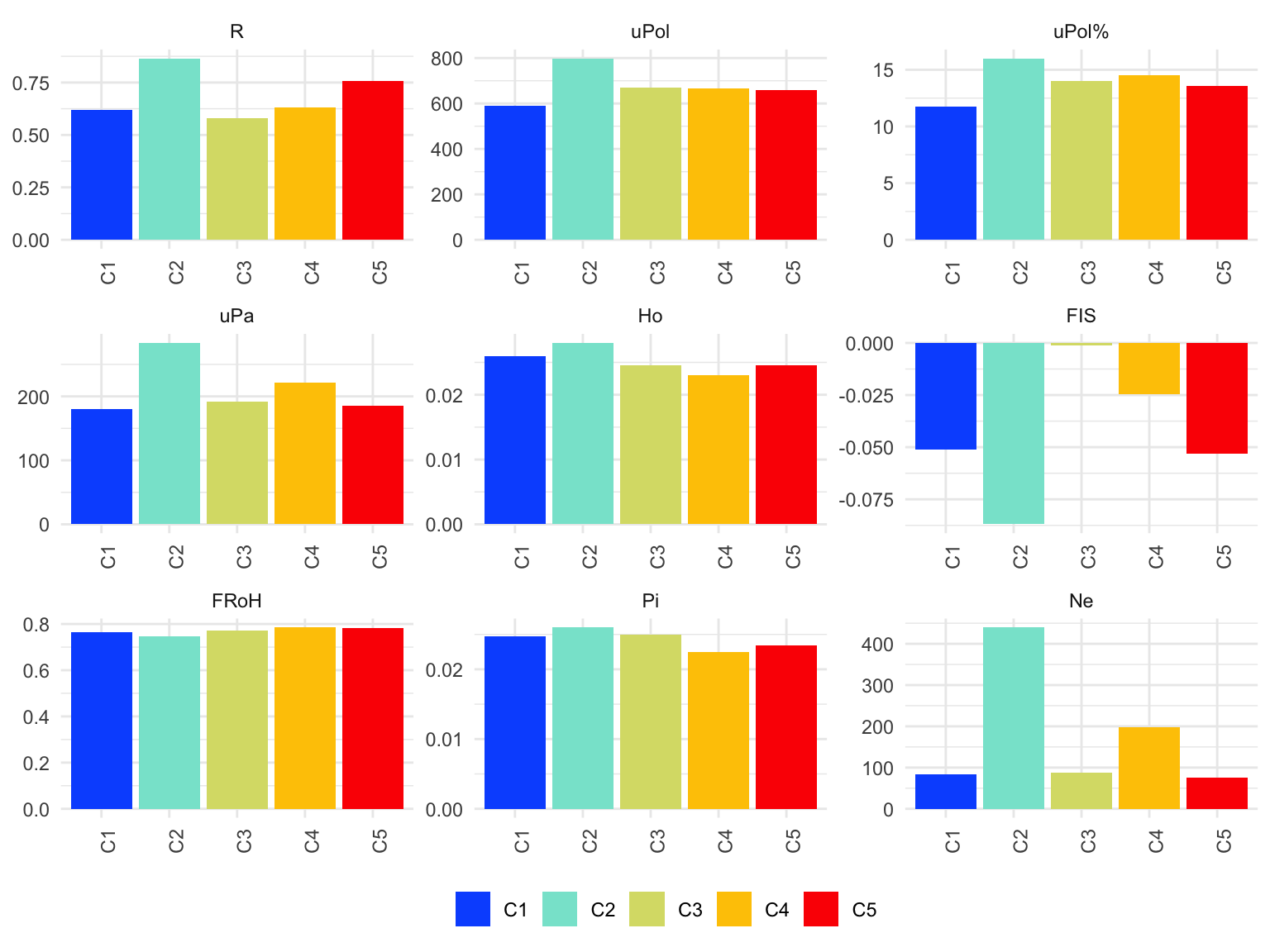


# *Fig S9: Meadow* genetic diversity estimates

*Mean genetic diversity estimates for the different meadows coloured by A) size and B) Impact*

A)
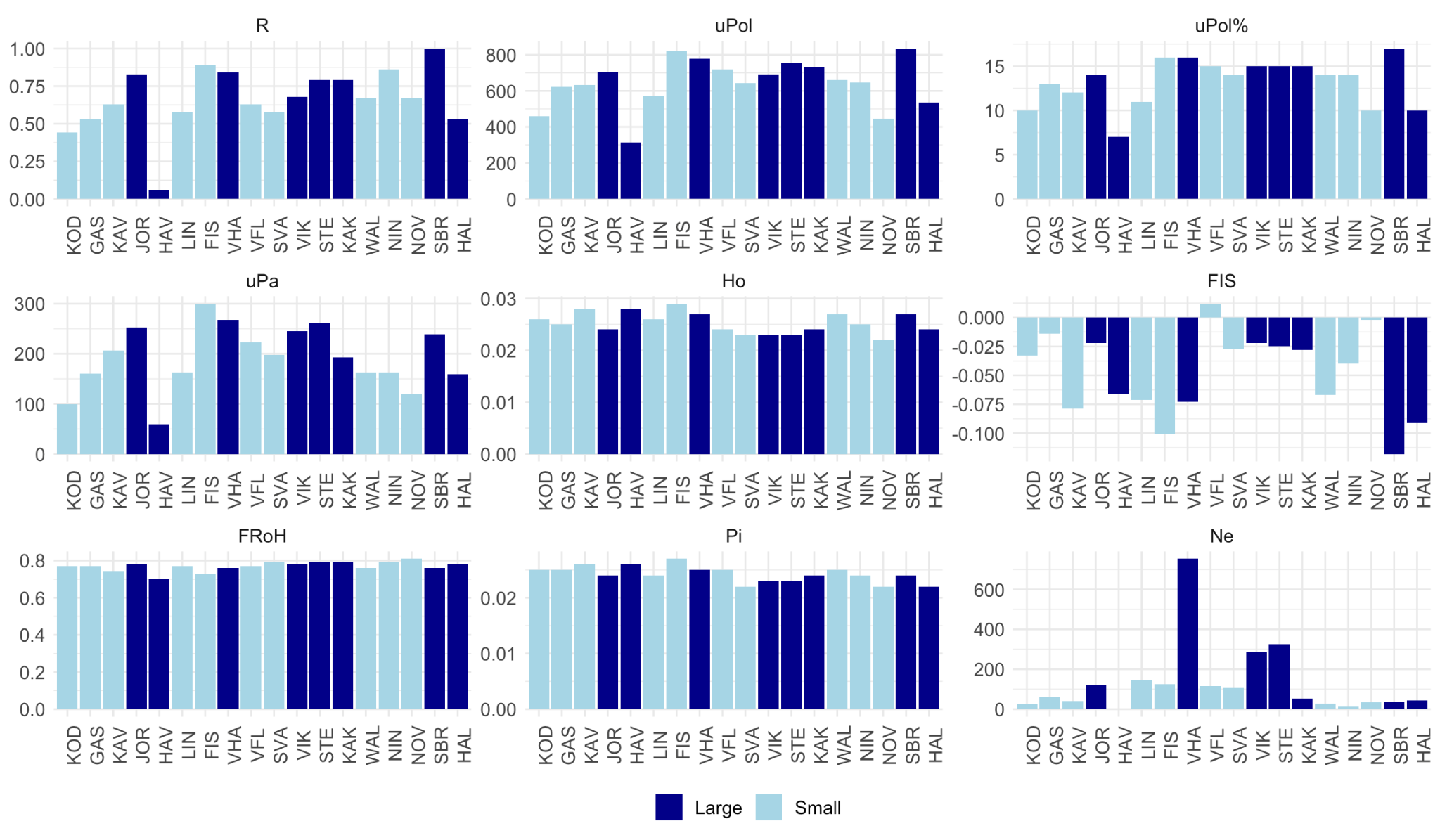


B)
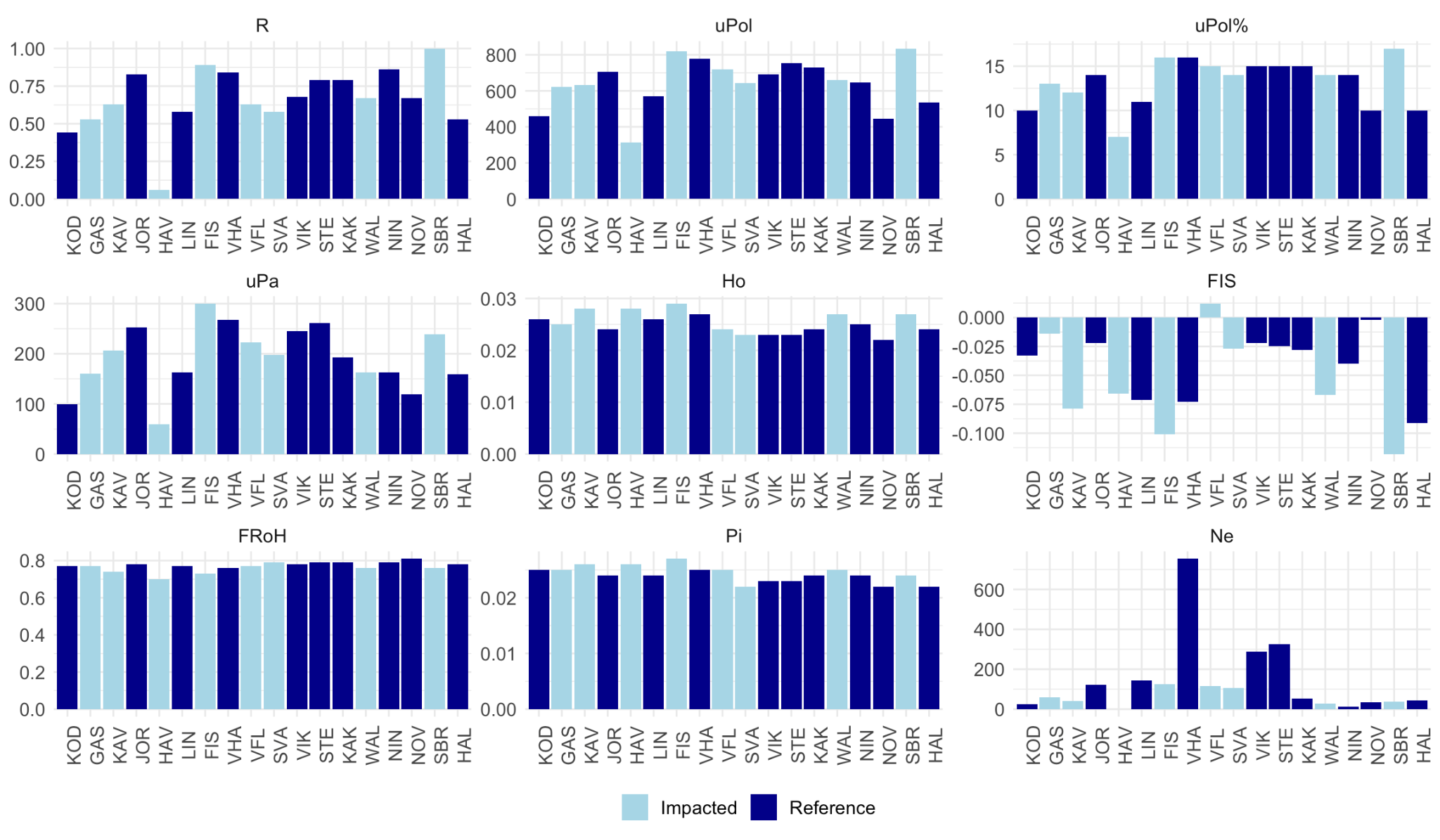


# Fig S10: ROHs

*Distribution of sizes of ROH in each individual. Top figure is prior to clone-correction and bottom is after clone-correction.*


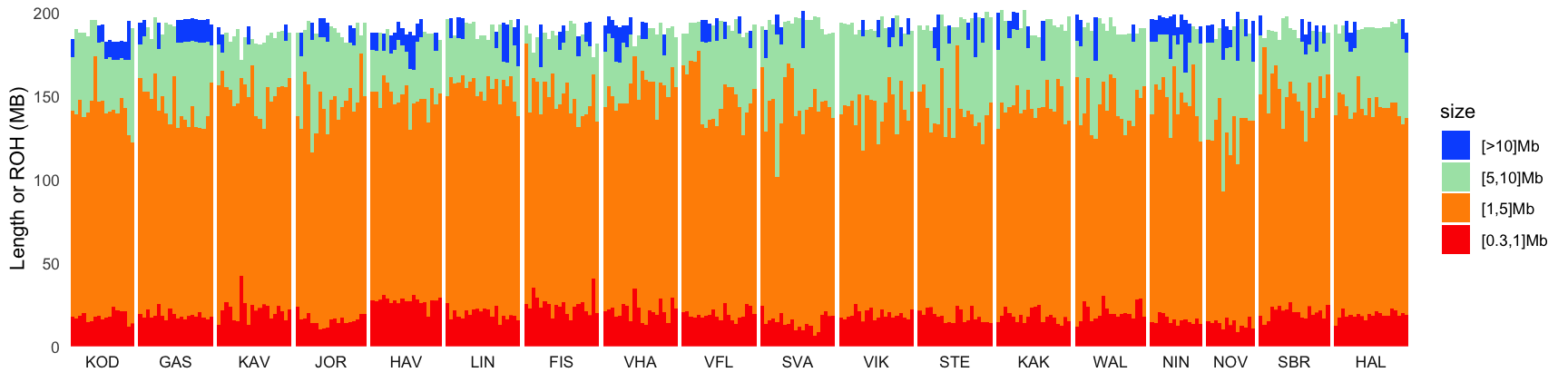


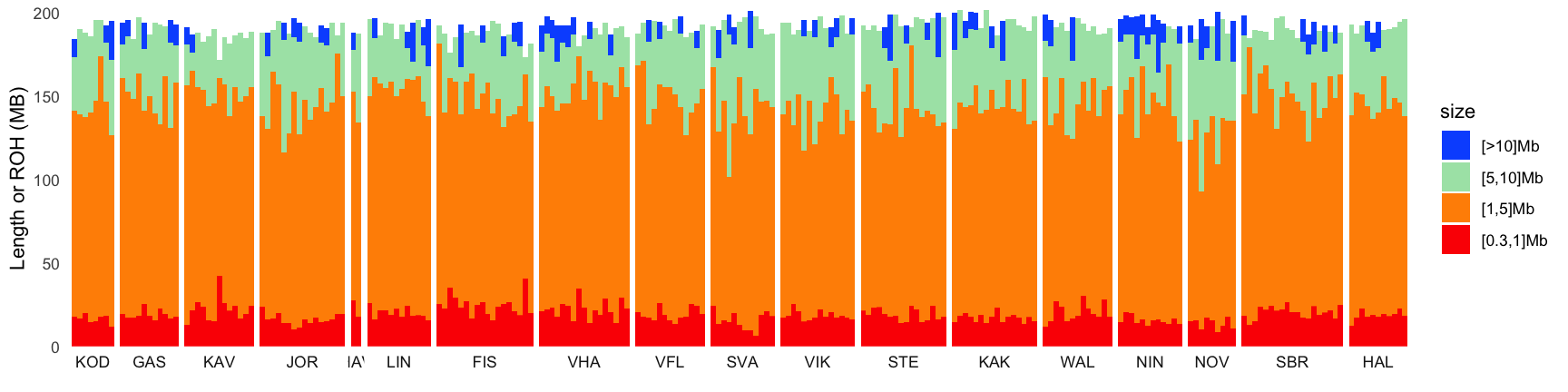


# Fig S11: FRoH

*Boxplots of individual inbreeding coefficients FRoH >0.3MB (top), FRoH >1Mb (middle) FRoH>5Mb (bottom). Left figures are prior to clone-correction and right are after clone-correction. Points represent individual values in each meadow.*
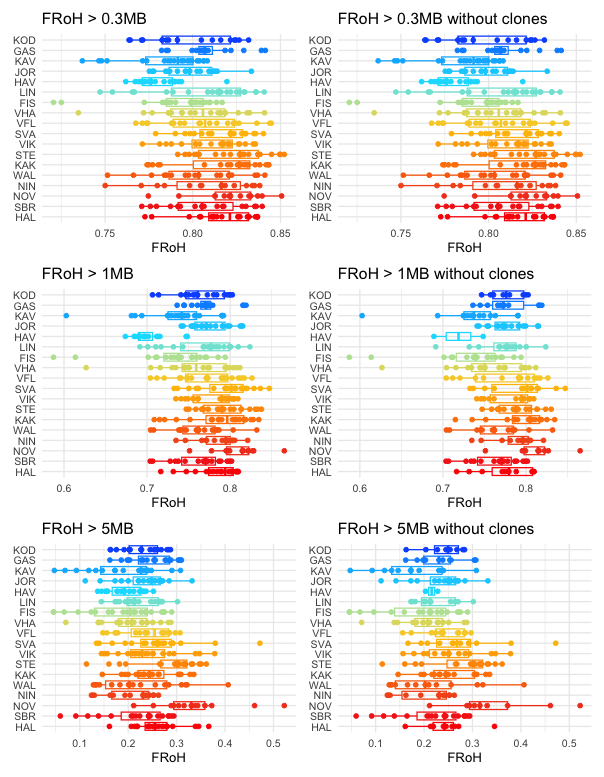


# Fig S12: Diversity and distribution correlation

Correlation between genetic diversity estimates and overall change in eelgrass areal distribution between 1980s to 2020s as a percentage per waterbody. A) Pearson's correlation coefficients with p-values and B) Spearman’s correlation coefficients with p-values.


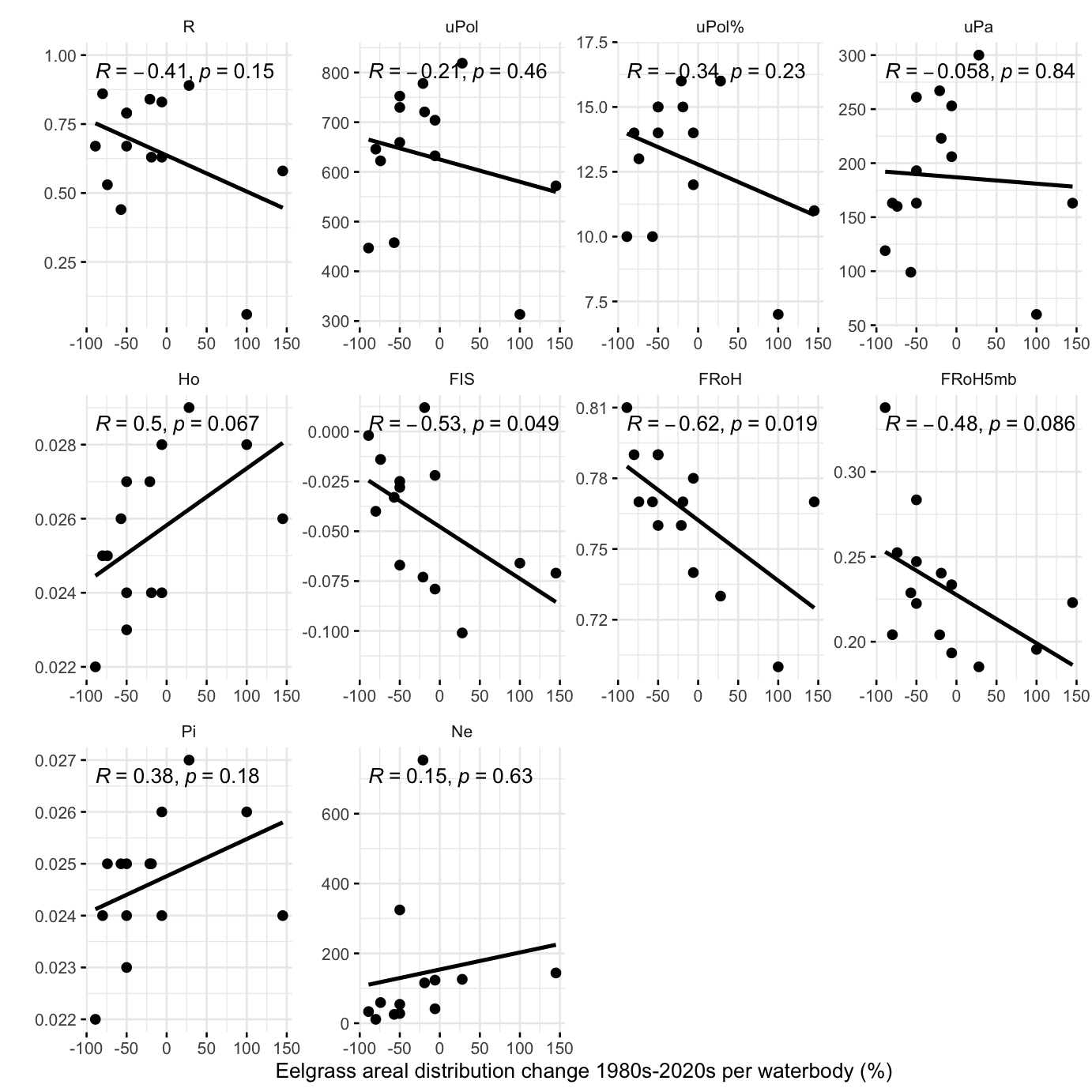


B)


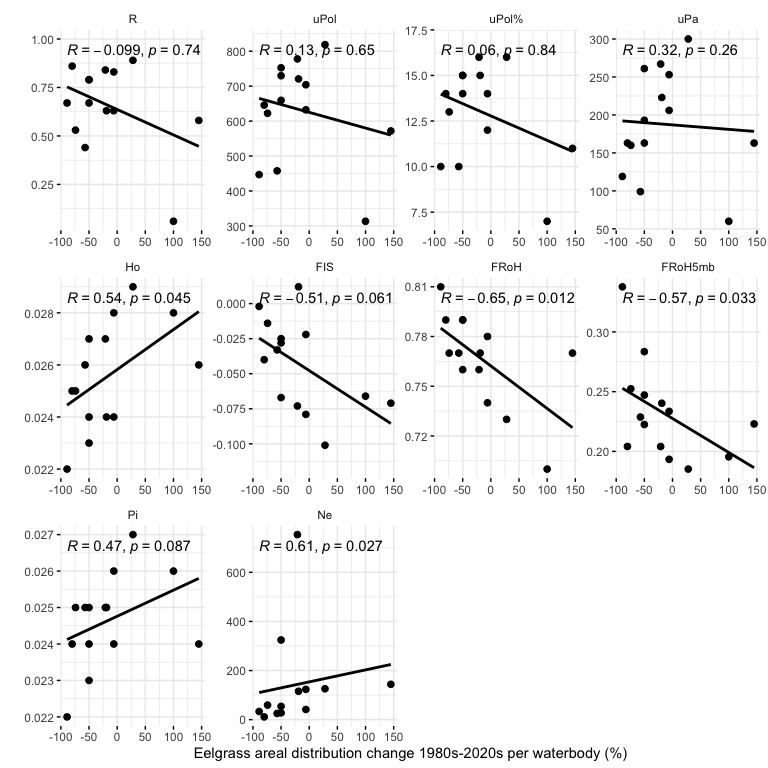


# *Fig S13:* Diversity and size correlation

Correlation between genetic diversity estimates and change in meadow size between 1980s to 2020s as a percentage.


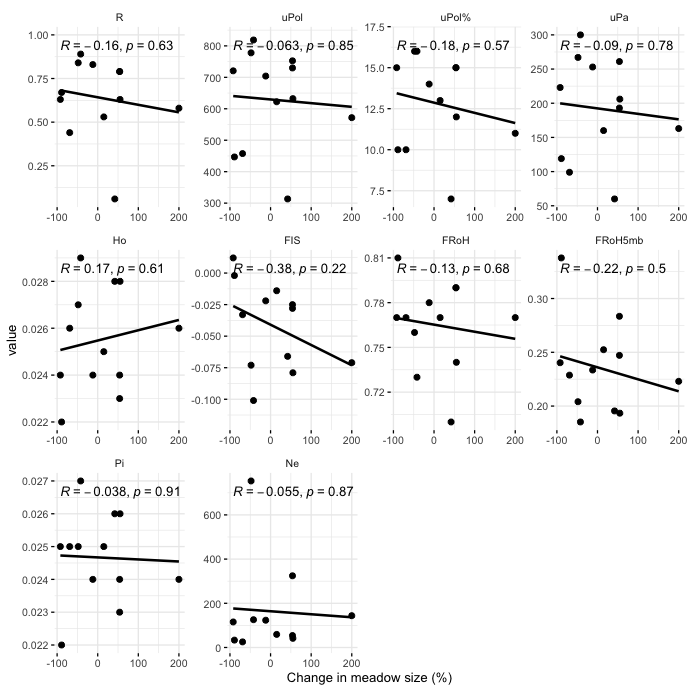


# Fig S14: Richness and waterbody change

*Correlations combining the data presented here with previous microsatellite analysis (Jahnke et al 2018; 2020). For the combined analyses genetic diversity was classified as low, medium and high based on either the unbiased percentage of polymorphic alleles (SNPs) or allelic richness (microsatellites). The different levels were then analysed separately, correlating genotypic richness (R) with overall change in eelgrass as a percentage per waterbody between the 1980s to 2020s.*


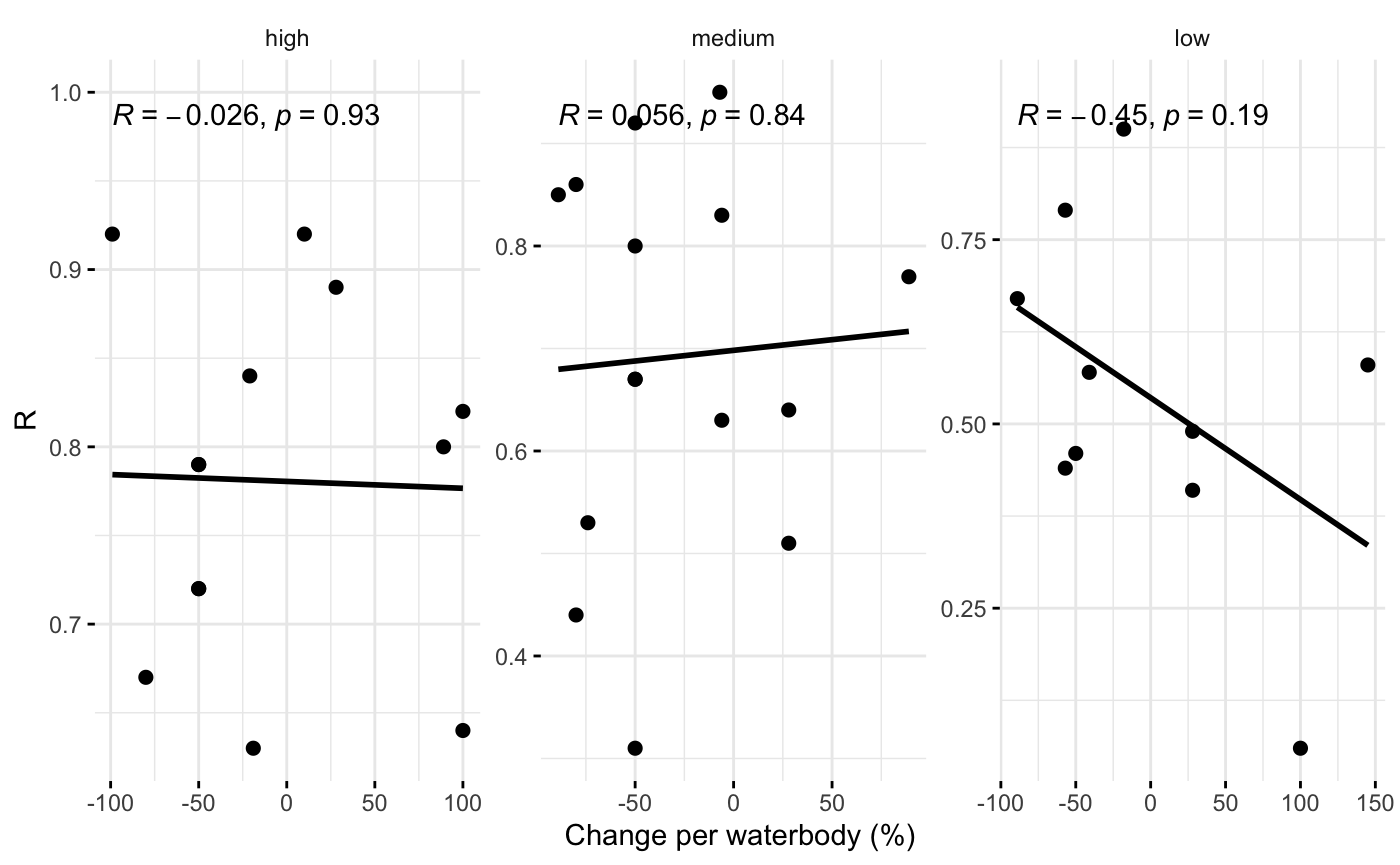

Supplement: Supplementary file 1 — Figure S1.–S14. [file MEC-34-e17656-s001.docx]
